# Cannabinoids Exacerbate Alcohol Teratogenesis by a CB1-Hedgehog Interaction

**DOI:** 10.1038/s41598-019-52336-w

**Published:** 2019-11-05

**Authors:** Eric W. Fish, Laura B. Murdaugh, Chengjin Zhang, Karen E. Boschen, Oswald Boa-Amponsem, Haley N. Mendoza-Romero, Michael Tarpley, Lhoucine Chdid, Somnath Mukhopadhyay, Gregory J. Cole, Kevin P. Williams, Scott E. Parnell

**Affiliations:** 1 Bowles Center for Alcohol Studies, University of North Carolina, Chapel Hill, NC USA; 2 Julius L. Chambers Biomedical/Biotechnology Research Institute, North Carolina Central University, Durham, NC USA; 3 Integrated Biosciences Program, North Carolina Central University, Durham, NC USA; 4 Biomanufacturing Research Institute and Technology Enterprise, North Carolina Central University, Durham, NC USA; 5 Department of Chemistry and Biochemistry, North Carolina Central University, Durham, NC USA; 6 Department of Biological and Biomedical Sciences, North Carolina Central University, Durham, NC USA; 7 Department of Pharmaceutical Sciences, BRITE Institute, North Carolina Central University, Durham, NC USA; 8 Department of Cell Biology and Physiology, University of North Carolina, Chapel Hill, NC USA; 9 Carolina Institute for Developmental Disabilities, University of North Carolina, Chapel Hill, NC USA

**Keywords:** Disease model, Development of the nervous system, Neurodevelopmental disorders

## Abstract

We tested whether cannabinoids (CBs) potentiate alcohol-induced birth defects in mice and zebrafish, and explored the underlying pathogenic mechanisms on Sonic Hedgehog (Shh) signaling. The CBs, Δ^9^-THC, cannabidiol, HU-210, and CP 55,940 caused alcohol-like effects on craniofacial and brain development, phenocopying Shh mutations. Combined exposure to even low doses of alcohol with THC, HU-210, or CP 55,940 caused a greater incidence of birth defects, particularly of the eyes, than did either treatment alone. Consistent with the hypothesis that these defects are caused by deficient Shh, we found that CBs reduced Shh signaling by inhibiting Smoothened (Smo), while *Shh* mRNA or a CB1 receptor antagonist attenuated CB-induced birth defects. Proximity ligation experiments identified novel CB1-Smo heteromers, suggesting allosteric CB1-Smo interactions. In addition to raising concerns about the safety of cannabinoid and alcohol exposure during early embryonic development, this study establishes a novel link between two distinct signaling pathways and has widespread implications for development, as well as diseases such as addiction and cancer.

## Introduction

Fetal Alcohol Spectrum Disorder (FASD) is common; recent conservative prevalence rates range from 1–5%^1^. Binge alcohol drinking (i.e. more than 4 or 5 drinks in two hours for women and men, respectively) is increasingly popular among young people, and is especially damaging to the embryo during the third to fourth weeks of pregnancy, when most pregnancies are unrecognized^2,3^. Alcohol exposure during this period causes characteristic craniofacial malformations of Fetal Alcohol Syndrome (FAS) including small palpebral fissures (micropthalmia), a smooth philtrum, and brain malformations in the holoprosencephaly spectrum^3^.

Marijuana use is also rising^4^, and about 4% of pregnancies are marijuana-exposed^5^, through either recreational use or as an anti-nausea self-medication. However, some subgroups of pregnancies have considerably more exposure^6,7^ and nearly 20% of a cohort of pregnant women in California, aged 18–24, report marijuana use^8^. As marijuana and other cannabinoids (CBs), such as cannabidiol (CBD), become increasingly legalized for medical or recreational purposes, and they remain perceived as low-risk substances^9^ safe to use during pregnancy, the incidence of CB-exposed pregnancies will rise even further. A recent study of births in Colorado found that the incidence of several birth defects has risen in the state during the period of marijuana legalization^10^.

CBs are a diverse family of compounds and in addition to the *Cannabis* derived CBs, include the synthetic CBs, which can be 100-fold more potent for CB receptors than is THC^11^, and the endocannabinoids (ECBs) which have several physiological functions, especially during development^12^. Perturbation of the ECB system by embryonic and/or fetal CB exposure has several developmental consequences. Before implantation, CBs can delay early embryo development and implantation^13^ and marijuana use has been associated with spontaneous abortion and pre-term birth, particularly when used with other drugs^14^. Limited studies in the 1970s and 1980s revealed that some prenatally-exposed individuals have physical and psychological differences resembling FASD^15,16^. Since today’s marijuana is at least 4-fold more potent^17^, it is likely that the consequences of prenatal CB exposure are more severe than first thought. Synthetic CBs, in particular, may pose greater harms to the embryo.

Animal studies, precisely controlling drug exposures, corroborate the morphological and life-long behavioral effects of both phyto- and synthetic-cannabinoids^18–23^. Previously, we found that a single, neurulation-stage exposure to the synthetic cannabinoid CP 55,940 dose-dependently induced craniofacial and brain abnormalities, mostly in the holoprosencephaly (HPE) spectrum^24^, and similar to a high-dose alcohol exposure^3^. During neurulation (gestational day [GD] 8–10 in the mouse, equivalent to the late third and early fourth weeks of human gestation), the neural tube closes, and the eyes and brain form out of the neuroepithelium. As predicted by these developmental events, CP 55,940 caused significant brain and eye defects, ranging from mild microphthalmia to severe anophthalmia, which are also common in FASD^25^. The sensitivity of the neurulation-stage embryo to many CBs supports clinical studies demonstrating that first trimester marijuana exposure, the most common exposure^7,26^, has developmental effects^27–29^.

CBs are frequently used with alcohol, partly to intensify their individual psychoactive effects, but also because one substance can disinhibit the use of the other^30,31^. Combined alcohol and CBs is significantly more impairing than is either substance alone and simultaneous use is associated with a greater risk for negative health outcomes^2,30–32^. Unfortunately, the combined effects of alcohol and CBs during pregnancy have not been systematically studied^33^. Boa-Amponsem *et al*.^34^ have recently demonstrated that combined exposure to a CB1 receptor agonist, ACEA, and ethanol worsen embryonic dysmorphology and juvenile behavioral phenotypes in zebrafish, in a manner reversible by either CB1 receptor antagonism or the addition of Sonic hedgehog (Shh) mRNA. Here, using mouse and zebrafish models of drug exposure during neurulation, we evaluated how simultaneous exposure to alcohol and a variety of CBs affects embryonic development and established a pathogenic mechanism involving a novel interaction between the CB1 receptor and the Shh signaling pathway. While prior studies implicate Shh signaling in alcohol teratogenesis^25,35–39^, less is known about how CBs affect Shh signaling. Recently, ECBs and phytocannabinoids (i.e. THC and CBD) were shown to inhibit Shh signaling *in vitro* by direct interactions with Smoothened (Smo)^40^. Smo, a G-protein-coupled, seven-pass transmembrane protein, regulates PKA levels and the activity of GPR161 within the primary cilium^41,42^ which affects the processing of downstream glioma associated oncogene (Gli) transcriptional activators. We now report that in the embryo, Smo and CB1 form heteromers that are likely targets of the teratogenic effects of simultaneous alcohol and CB exposure.

## Results

### Teratogenesis of prenatal cannabinoid exposure

**Figure 1:**
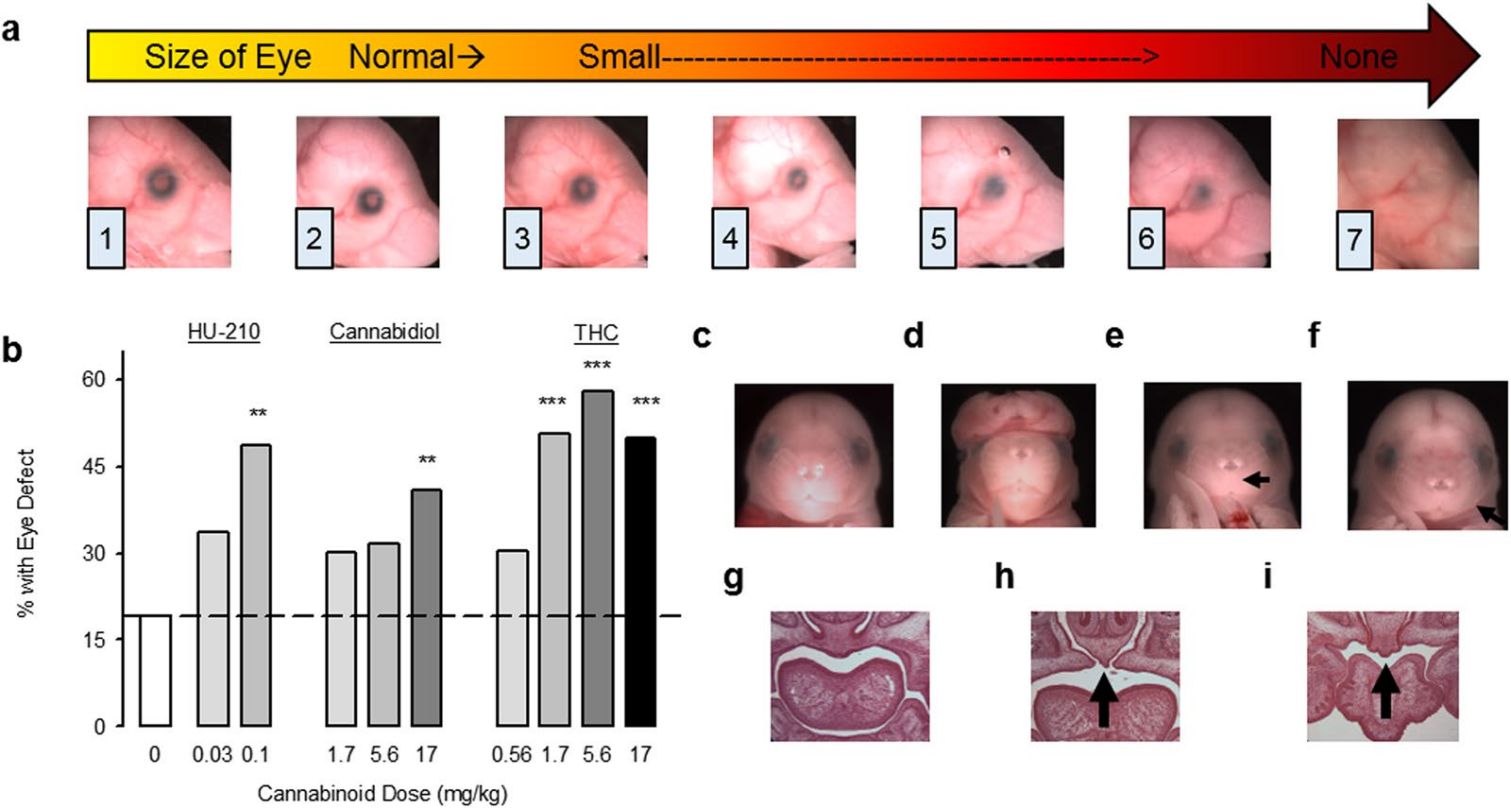
Prenatal cannabinoid exposure causes eye and face malformations in fetal mice. (**a**) Representative photographs of GD 17 mice that had been exposed to CB treatments on GD 8, with eyes ranked in severity from normal (1) to anophthalmic (7). Minor alterations of pupil shape and size occur in ranks (2) and (3), while colobomas are scored as (4–6). (**b**) The incidence of defects in either the right or the left eye in fetal mice following GD 8 exposure to the CB vehicle (n = 63 fetuses/8 litters), HU-210 0.03 mg/kg (n = 89 fetuses/11 litters), HU-210 0.1 mg/kg (n = 35 fetuses/6 litters), CBD 1.7 mg/kg (n = 63 fetuses/8 litters), CBD 5.6 mg/kg (n = 60 fetuses/7 litters), CBD 17 mg/kg (n = 78 fetuses/10 litters), THC 0.56 mg/kg (n = 76 fetuses/9 litters), THC 1.7 mg/kg (n = 81 fetuses/11 litters), THC 5.6 mg/kg (n = 69 fetuses/10 litters), THC 17 mg/kg (n = 80 fetuses/10 litters). **p < 0.01, ***p < 0.001, Chi-Square vs. CB vehicle. (**c–i**) Craniofacial and palate malformations caused by prenatal CBs. (**c**) A typical face following CB vehicle treatment. (**d**) An exencephaly caused by HU-210 (0.1 mg/kg, 4 cases). (**e**) A philtrum deficiency caused by CBD (17 mg/kg, 1 case). (**f**) micrognathia caused by THC (17 mg/kg, 1 case, also observed after CBD 1.7 and 5.6 mg/kg). (**g**) Hematoxylin and eosin staining of coronal sections through the anterior palate of a typical CB vehicle-treated fetus. (**h**,**i**) Cleft anterior palates caused by THC (**h** 1.7 mg/kg, **i** 17 mg/kg), as indicated by the black arrows. Data are expressed as the percent of mice with eye defects (number of affected mice/total number of mice *100), observed in a single experiment. See also Supplementary Table 1 for data on eye defect severity, body weight and length, and the number of live offspring per litter. See Supplementary Fig. 2a,b for separate analysis of left and right eye defects.

We first extended our original findings from CP 55,940^24^ to other CBs. Pregnant C57 mice received a single intraperitoneal injection of the synthetic cannabinoid HU-210, the phytocannabinoids CBD, or Δ^9^-THC, or the CB vehicle on their 8^th^ day of pregnancy (GD 8 – the beginning of neurulation). While THC concentrations vary between cannabis strains and preparation, the mouse THC doses (0.56–17.0 mg/kg) replicate peak blood levels in the range that frequent cannabis users can achieve in a controlled laboratory setting^43–49^. The CBD doses administered in this study (1.7–17 mg/kg) are within the therapeutic range (<1–50 mg/kg/day) for several medical conditions^50^. Eye defects were evaluated using a dysmorphology scale modified from^24,51^ (Fig. 1a). This qualitative method was previously found to better distinguish between affected and unaffected eyes than was computer-based ocular measurement because of the ability to assess both size and shape deviations^51^. Each CB dose-dependently increased the eye defect incidence above that following vehicle injections (Fig. 1b). In contrast to normal craniofacies (Fig. 1c,g), gross dysmorphologies, including exencephaly (Fig. 1d), philtrum deficiency (Fig. 1e), and small mandibles (agnathia; Fig. 1f), were also observed following CB exposure. Anterior palate clefts, ranging from minor to quite severe (Fig. 1h,i), were evident in two THC-treated mice chosen for histological sectioning because of their dysmorphic eyes. All CBs dose-dependently reduced fetal body weight (Supplemental Table 1), an effect observed clinically^16,33^. While the CBs were mostly similar, HU-210, the most potent and longest acting compound in our studies, had the largest body weight decrease, reduced litter size, although the latter effect was not statistically significant (Supplemental Table 1), and caused the highest incidence of severe dysmorphology. In terms of overall percent of affected fetuses, CBD was the least teratogenic CB, causing ~20% increase above vehicle at the highest dose (17 mg/kg). However, the highest dose induced several severe eye defects (Supplemental Table 1) and a philtrum deficiency, characteristic of FAS. The difference between CBD and the other CBs may be dose-related (i.e. CBD may require higher doses to affect many mice) or mechanism-related. Unlike HU-210 and THC, CBD is not a CB1 receptor agonist, but has several actions^52^, including affecting Shh signaling, at least *in vitro*^40^. The teratology studies demonstrate that eyes are sensitive to prenatal CB exposures, but because at GD 8 the eyes develop from the same neuroepithelium as the brain, we also observed numerous severe brain dysmorphologies, ranging from exencephaly to holoprosencephaly. To investigate excessive neuroepithelium cell death, we examined apoptosis following moderately high exposure to CP 55,940 (0.5 mg/kg), the CB1 agonist examined in our previous teratology study^24^, because its effects are highly dose-dependent without being embryonically lethal. CP 55,940 did not increase embryonic cell death, ruling this out as a primary pathogenic mechanism (Supplemental Fig. 1).

### Prenatal cannabinoid exposure potentiates the effects of alcohol in mice and zebrafish

**Figure 2:**
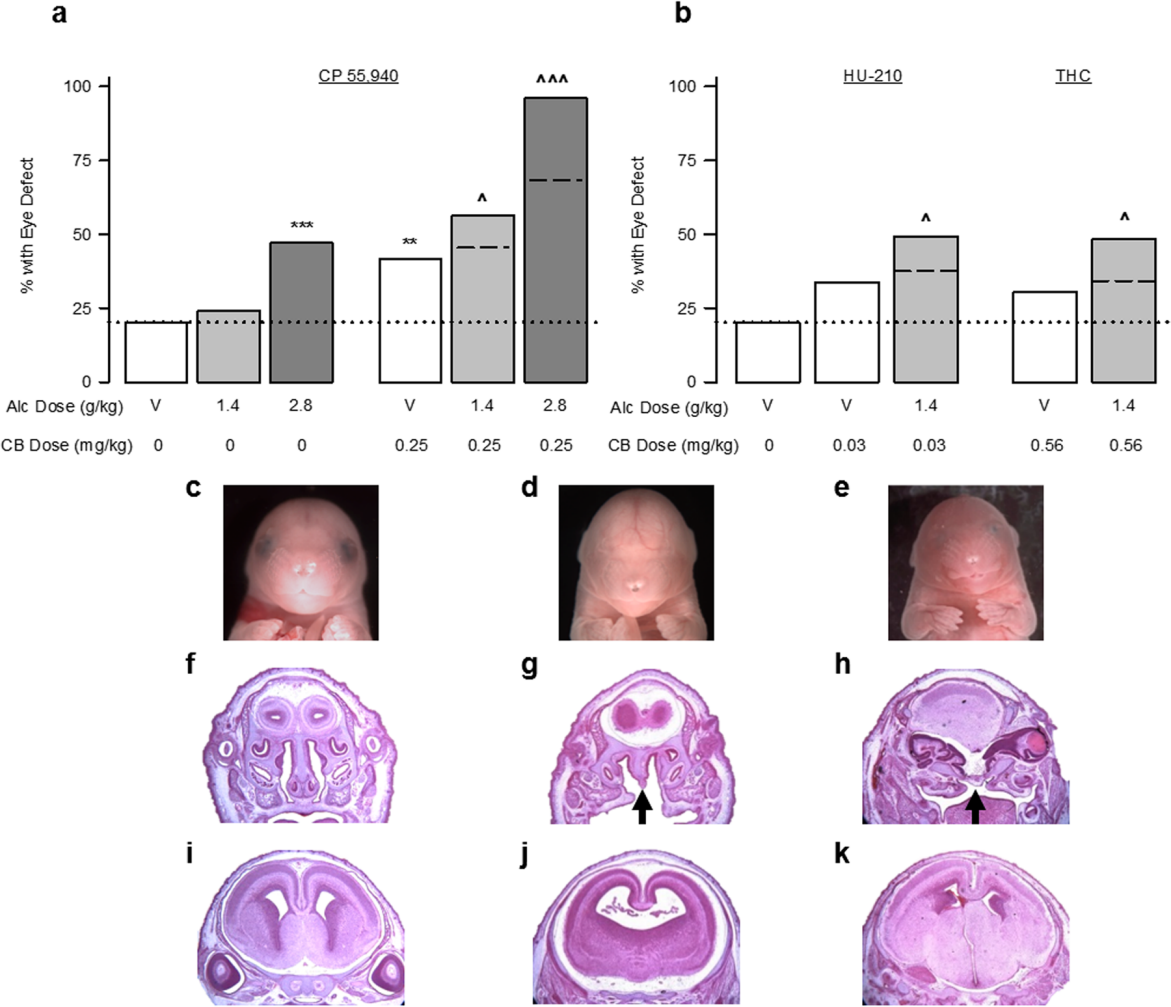
Cannabinoids exacerbate alcohol-induced eye and face malformations. (**a**) The incidence of defects in either the right or the left eye in fetal mice following GD 8 exposure to the CB vehicle (n = 103 fetuses/13 litters), Alcohol 1.4 g/kg alone (n = 62 fetuses/10 litters), Alcohol 2.8 g/kg alone (n = 85 fetuses/10 litters), CP 55,940 0.25 mg/kg alone (n = 101 fetuses/12 litters), simultaneous CP 55,940 0.25 mg/kg + Alcohol 1.4 g/kg (n = 133 fetuses/16 litters), or simultaneous CP 55,940 0.25 mg/kg + Alcohol 2.8 g/kg (n = 50 fetuses/7 litters). (**b**) The incidence of defects in either the right or the left eye in fetal mice following GD 8 exposure to the CB vehicle (n = 103 fetuses/13 litters), HU-210 0.03 mg/kg alone (n = 89 fetuses/11 litters), simultaneous HU-210 0.03 mg/kg + Alcohol 1.4 g/kg (n = 122 fetuses/15 litters), THC 0.56 mg/kg alone (n = 76 fetuses/9 litters), or simultaneous THC 0.56 + Alcohol 1.4 g/kg (n = 87 fetuses/11 litters). Data are expressed as the percent of mice with eye defects (number of affected mice/total number of mice *100), observed in a single experiment. **p < 0.01, ***p < 0.001, vs CB vehicle; ^p < 0.05, ^^^p < 0.001 vs. respective doses of CP 55,940 alone using a Chi-square test. (**c–k**) Craniofacial, palate, and brain malformations following simultaneous alcohol and cannabinoids. (**c,f,i**) A typical fetal mouse face as well as hematoxylin and eosin staining of coronal sections through the anterior palate and the brain at the level of the septal area, following the CB vehicle treatment. (**d,g,j**) Anophthalmia and philtrum deficiency, as well as cleft palate (indicated by black arrows), holoprosencephaly, and septal deficiency caused by alcohol 1.4 g/kg + CP 55,940 0.25 mg/kg. (**e,h,k**) Near anophthalmia, philtrum, and nostril deficiency, as well as cleft palate and widespread brain structural changes caused by alcohol 1.4 g/kg + HU-210 0.03 mg/kg. See also Supplementary Table 2 for data on eye defect severity, body weight and length, and the number of live offspring per litter. See Supplementary Fig. 2c–f for separate analysis of left and right eye defects.

We next combined CB and alcohol exposure, first in the mouse model described above, and then in a zebrafish model. We again focused on the synthetic CB1 receptor agonist CP 55,940 because of, as mentioned above, its dose-dependent and non-cytotoxic effects. In the mouse, alcohol dose-dependently increased the incidence of eye defects (Fig. 2a) and a moderate CP 55,940 dose (0.25 mg/kg) alone was equally deleterious (Fig. 2a). Combining CP 55,940 with either the low (1.4 g/kg) or high (2.8 g/kg) alcohol doses significantly increased eye defects above the incidence caused by either treatment alone. The increase was greater than a predicted additive effect (10.9% greater for the low alcohol dose and 27.6% greater for the high alcohol dose). CP 55,940 did not affect blood alcohol levels following the low alcohol dose (Supplemental Table 3). We confirmed that other CBs potentiate alcohol teratogenesis by examining co-exposure to alcohol and HU-210 (0.03 mg/kg) or THC (0.56 mg/kg) dosages that do not significantly induce dysmorphologies. When given with alcohol (1.4 g/kg), HU-210 and THC significantly increased the eye defect incidence, relative to either CB treatment alone, exceeding the predicted additive effect by 11.6% and 14.0%, for HU-210 and THC respectively (Fig. 2b). Severe craniofacial and brain dysmorphologies were noted after the combined treatments that were not evident following either drug alone. Compared to a vehicle-treated fetus (Fig. 2c,f,i), severe eye defects (complete and partial anophthalmia), philtrum deficiencies, holoprosencephaly, and cleft palates are shown from a fetus treated with low dose alcohol and CP 55,940 (Fig. 2d,g,j) and a fetus treated with low dose alcohol and HU-210 (Fig. 2e,h,k).

**Figure 3:**
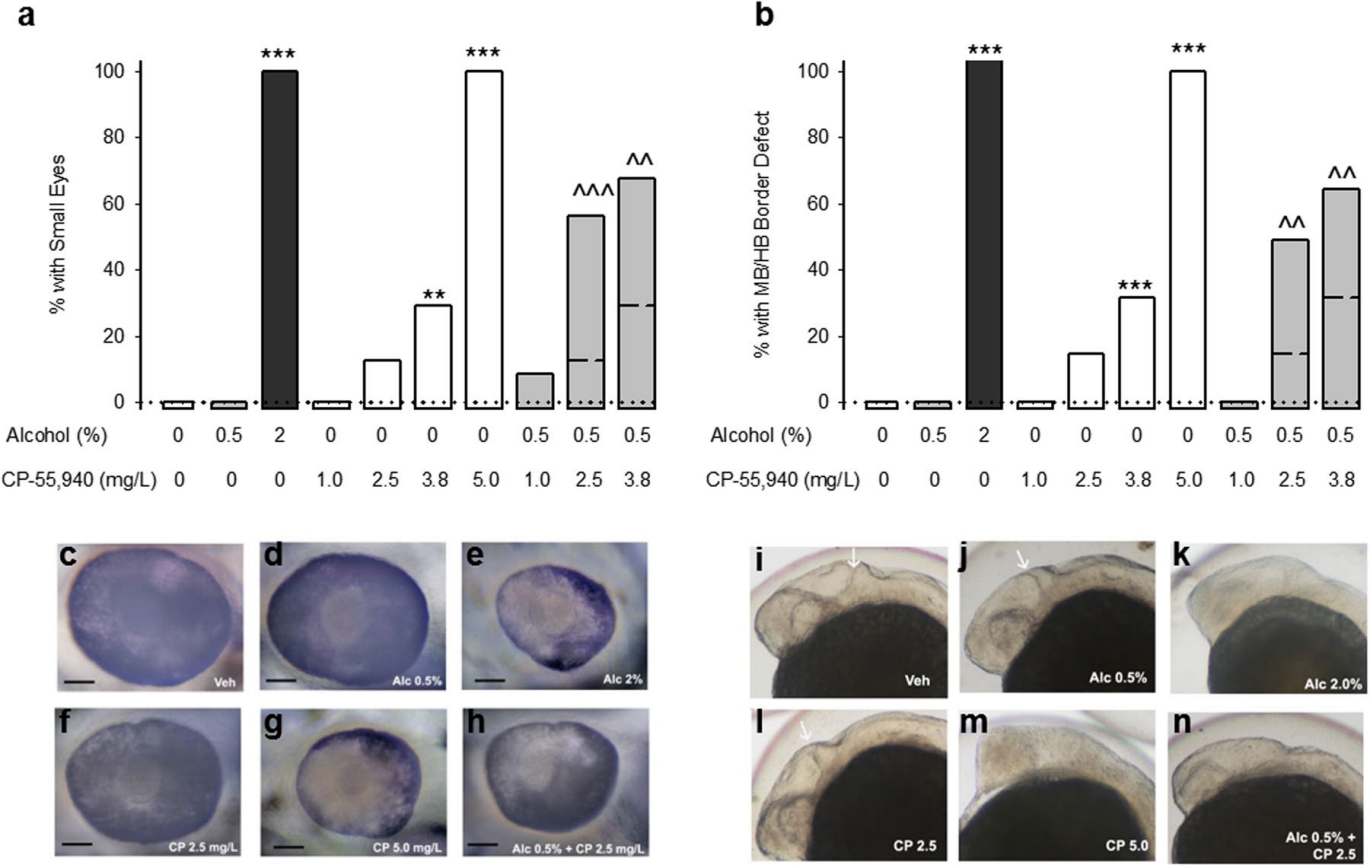
Cannabinoids exacerbate alcohol-induced eye and brain malformations in zebrafish. (**a,b**) The incidence of small eyes (**a**) and midbrain/hindbrain boundary defects (**b**) following exposure to fish water vehicle (n = 30 for eyes and MHB), 0.5% alcohol (n = 30 for eyes and MHB), 2.0% alcohol (n = 30 for eyes and MHB), CP 55,940 1.0 mg/L (n = 30 for eyes and MHB), CP 55,940 2.5 mg/L (n = 32 for eye, 34 for MHB), CP 55,940 3.8 mg/L (n = 41 for eye, 44 for MHB), CP 55,940 5.0 mg/L (n = 40 for eye, 43 for MHB), 0.5% alcohol + CP 55,940 1.0 mg/L (n = 32 for eye, 35 for MHB), 0.5% alcohol + CP 55,940 2.5 mg/L (n = 35 for eye, 45 for MHB), 0.5% alcohol + CP 55,940 3.8 mg/L (n = 44 for eye, 49 for MHB). MHB defects were measured following exposure from 5.25 to 24 hours post fertilization (hpf), and small eyes were measured following exposure from 5.25 to 48 hpf. **p < 0.01, ***p < 0.001, vs fish water vehicle, ^^p < 0.01, ^^^p < 0.001 vs. respective doses of CP 55,940 alone using a Chi-square or Fischer’s exact test, depending on sample size. Data are expressed as the percent of zebrafish with defects (number of affected zebrafish/total number of zebrafish *100), observed in a single experiment. Dotted lines reference the zero incidence of spontaneous eye and MHB defects. Dashed lines within the bars corresponding to the alcohol and CP 55,940 simultaneous treatment indicate the predicted additive effects. (**c–h**) Representative photographs of zebrafish eyes and midbrain/hindbrain boundaries (**i**–**n**) following vehicle (**c,i**), 0.5% alcohol (**d,j**), 2% alcohol (**e,k**), CP 55,940 2.5 mg/L (**f,l**), CP 55,940 5.0 mg/L (**g** and **m**), and 0.5% alcohol + CP 55,940 2.5 mg/L (**h** and **n**). The black line in (**c–h**) represents a 50 μm scale bar. The white arrows in (**i,j,l**) indicate the midbrain/hindbrain boundary.

Zebrafish phenocopy the effects of alcohol observed in humans and mice^53^, and our zebrafish studies herein confirmed that CBs induce birth defects on their own and potentiate alcohol teratogenesis. Alcohol or CP 55,940 dose-dependently increased microphthalmia (Fig. 3a,c–h for representative images) and midbrain/hindbrain boundary defects (MHB, Fig. 3b,i–n for representative images) examined 24 or 48 hours after the start of the exposure. Combining CP 55,940 (1–3.8 mg/L) with an alcohol concentration (0.5%) that is not grossly teratogenic, potentiated the incidence of microphthalmia and MHB defects relative to the CP 55,940 treatment alone. This cross-species conservation of teratogenesis further demonstrates the potential danger of these two drugs and implicates a conserved mechanism of action.

### Cannabinoid inhibition of the Sonic Hedgehog pathway is a pathogenic mechanism

**Figure 4:**
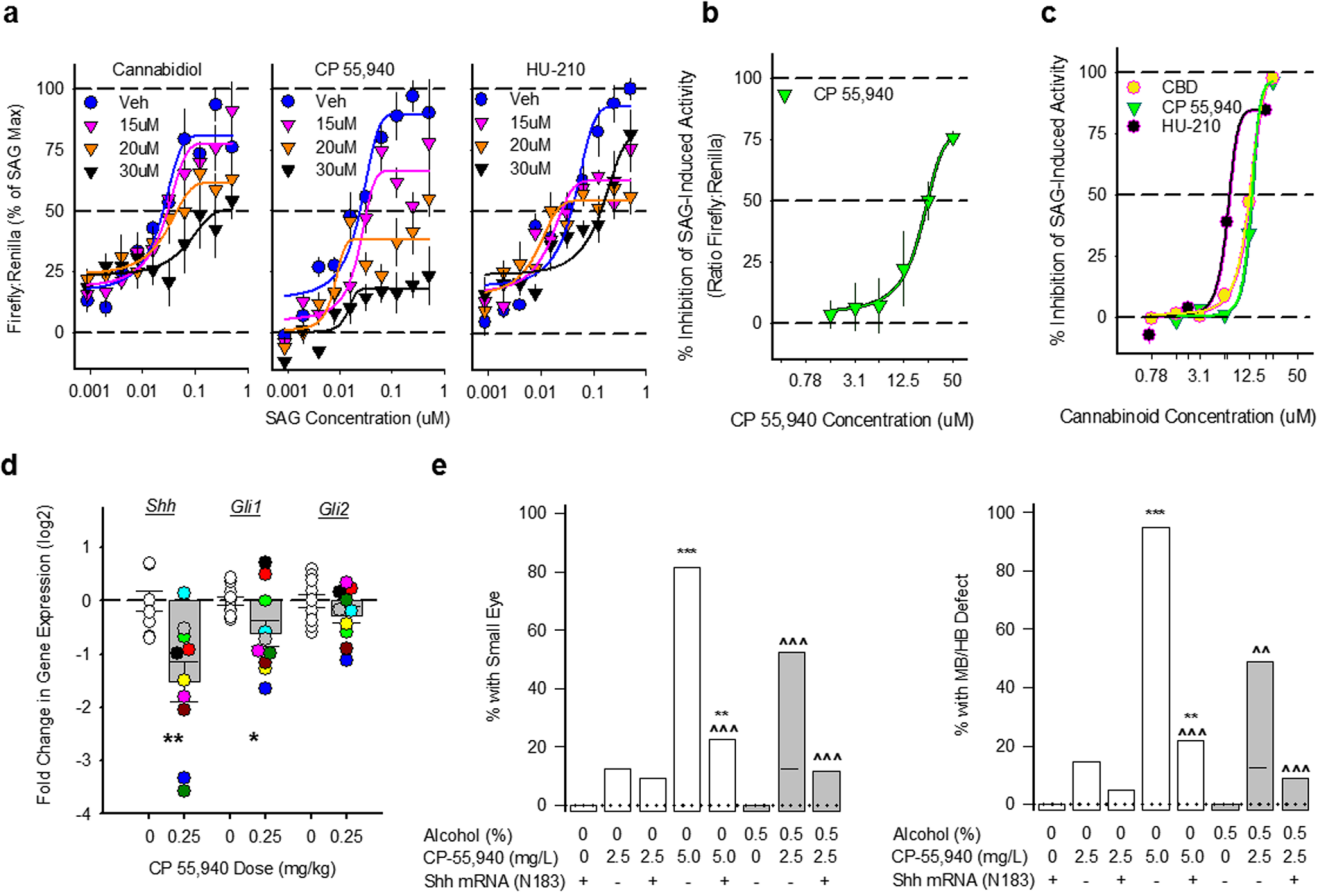
Cannabinoids impair Sonic Hedgehog signaling. (**a**) Effects of different concentrations of CBD, CP 55,940 and HU-210 on Smoothened agonist, SAG, stimulation of Gli1-mediated firefly luciferase expression in ShhLight2 cells. All data are the mean (±SEM) ratio of Gli1-mediated expression of firefly luciferase to constitutive renilla luciferase from 6 samples conducted in triplicate in two independent experiments, normalized as a percent inhibition of the maximal SAG effect. (**b**) Effects of different concentrations of CP 55,940 on Gli1-mediated firefly luciferase induced by a maximally effective SAG concentration (300 nM) in ShhLight2 cells. All data are the mean (±SEM) ratio of Gli1-mediated expression of firefly luciferase to constitutive renilla luciferase from 5 samples performed in triplicate in a single experiment. (**c**) Effects of different concentrations of CBD (yellow), CP 55,940 (green) and HU-210 (black) on Gli1-mediated alkaline phosphatase expression induced by a maximally effective SAG concentration (100 nM) in C3H10T1/2 cells. All data are mean (±SEM) alkaline phosphatase expression from 3 replicate samples from a single experiment, normalized as a percent inhibition of the SAG effect. (**d**) Shh pathway gene expression as measured by qRT-PCR in mouse rostral neural tube, 24 hours following GD 8 exposure to the CB vehicle or CP 55,940 (0.25 mg/kg). *Shh*, *Gli1*, and *Gli2* are normalized to the housekeeping gene *18 s* and portrayed as log_2_ fold change from vehicle. All samples were measured in triplicate and the data are portrayed from a single experiment. Individual embryos are portrayed as open or filled circles (n = 9 embryos/5 litters for vehicle for *Shh*, or 10 embryos/5 litters for *Gli1* and *Gli2* vehicle; n = 10 embryos/4 litters for CP 55,940), while means (±SEM) are portrayed as open or filled bars. *p < 0.05, **p < 0.01 using two-sided Student’s t-tests. Individual embryos are marked by different fill colors. (**e**) The incidence of small eyes and midbrain/hindbrain boundary defects in zebrafish embryos receiving exposure to Shh mRNA (N183) + fish water vehicle (n = 40 for eye, 45 for MHB), CP 55,940 2.5 mg/L alone (n = 32 for eye, 34 for MHB), Shh mRNA (N183) + CP 55,940 2.5 mg/L (n = 32 for eye, 41 for MHB), CP 55,940 5.0 mg/L alone (n = 54 for eye, 57 for MHB), Shh mRNA (N183) + CP 55,940 5.0 mg/L (n = 53 for eye, 46 for MHB), 0.5% alcohol alone (n = 41 for eye, 45 for MHB), Shh mRNA (N183) + 0.5% alcohol (n = 30 for eye and MHB), 0.5% alcohol + CP 55,940 2.5 mg/L (n = 35 for eye, 45 for MHB), Shh mRNA (N183) + 0.5% alcohol + CP 55,940 2.5 mg/L (n = 34 for eye, 45 for MHB). **p < 0.01, ***p < 0.001 vs Shh mRNA (N183) + vehicle, ^^p < 0.01, ^^^p < 0.001 vs corresponding CP 55,940 treatment in absence of Shh mRNA using a Chi-square test. Data are expressed as the percent of zebrafish with defects (number of affected zebrafish/total number of zebrafish *100) from a single experiment.

The craniofacial and brain defects observed in both species are qualitatively similar to impaired Shh signaling, providing a clue about pathogenic mechanisms. To examine whether synthetic CBs inhibit Shh signaling *in vitro*, as do CBD, THC, and endocannabinoids^40^, we first utilized Shh-Light2 cells, an engineered GLI-reporter line, which constitutively express renilla luciferase, indicating background cellular activity, and specifically express firefly luciferase upon Hh pathway activation. Varying concentrations of the Smo agonist, SAG, stimulated firefly luciferase expression. CP 55,940, HU-210, or CBD dose-dependently shifted the SAG concentration-response curves downward, indicating Shh pathway inhibition (Fig. 4a). The CBD experiment confirms previous findings^40^, with our CP 55,940 and HU-210 experiments indicating that Shh signaling inhibition may also be a mechanism for synthetic CBs. CBD and HU-210 reduced renilla expression, but downward shifts in the SAG concentration-response were still evident in the firefly:renilla ratio. HU-210 was the least effective when corrected for renilla, suggesting that unlike CP 55,940, it is cytotoxic at very high concentrations, which is consistent with our mouse teratology findings. We assessed overt cellular toxicity by staining the Shh2Light cells with Hoechst and YOYO-1 stains and found the threshold for CP-55,940, HU-210, and CBD to be 50 uM, which is higher than the 30 uM concentrations used in the current experiment (Supplementary Figs. 3 and 4). To further test how CP 55,940 affects SAG-induced activation of Gli1, we stimulated with a fixed, maximally effective SAG concentration (300 nM) and varied the CP 55,940 concentrations. CP 55,940 inhibited maximal SAG-activation, without affecting renilla expression (Fig. 4b). We repeated this experiment in a different cell line model, C3H10T1/2 cells which express alkaline phosphatase upon Hh/Gli1 activation. As in the ShhLight2 cells, CP 55,940, HU-210, and CBD concentration-dependently inhibited SAG (100 nM) activation of alkaline phosphatase (Fig. 4c). No CB showed any evidence for cytotoxicity in this cell line, which may account for the apparent greater efficacy in the C3H10T1/2 cells vs. the ShhLight2 cells. Taken together, these experiments strongly support the hypothesis that CBs can inhibit Shh signaling. That CBD was equally efficacious and equipotent to HU-210 and CP 55,940 suggests that effects on Shh signaling are not solely mediated by CB1 receptor agonism.

We next tested if CP 55,940 inhibits Shh signaling in the mouse embryo. We focused on CP 55,940 because it was teratogenic and inhibited Shh activity without the *in vitro* cytotoxicity of the other CBs. Twenty-four hours after a GD 8 CP 55,940 (0.25 mg/kg) exposure, we collected embryos with 15–16 somites, dissected out the rostral neural tube (which will form the brain and the face), and processed them individually with real-time quantitative PCR for the expression of *Shh*, *Gli1*, and *Gli2* mRNA, relative to the housekeeping gene *18s*. CP 55,940 significantly reduced *Shh* and *Gli1* gene expression, relative to stage-matched vehicle-treated embryos (Fig. 4d).

In chick and zebrafish embryos, increasing Shh prevents alcohol teratogenesis^35,54^. To investigate if this same phenomenon would occur following CB exposure, we injected one to two cell stage zebrafish embryos with ShhN183 mRNA before CP 55,940 and/or alcohol exposure (2.5 mg/L and 0.5%, respectively). This amount of N183 mRNA had no detectable effects on eye or brain morphology when given alone (Fig. 4e), but blocked the effects of CP 55,940, and the combined effects of alcohol and CP 55,940, on microphthalmia and the MHB border (Fig. 4e). These data demonstrate that the teratogenic mechanisms of alcohol and CBs converge through the Shh signaling pathway.

### The CB1 receptor also mediates the teratogenic effects of cannabinoids

**Figure 5:**
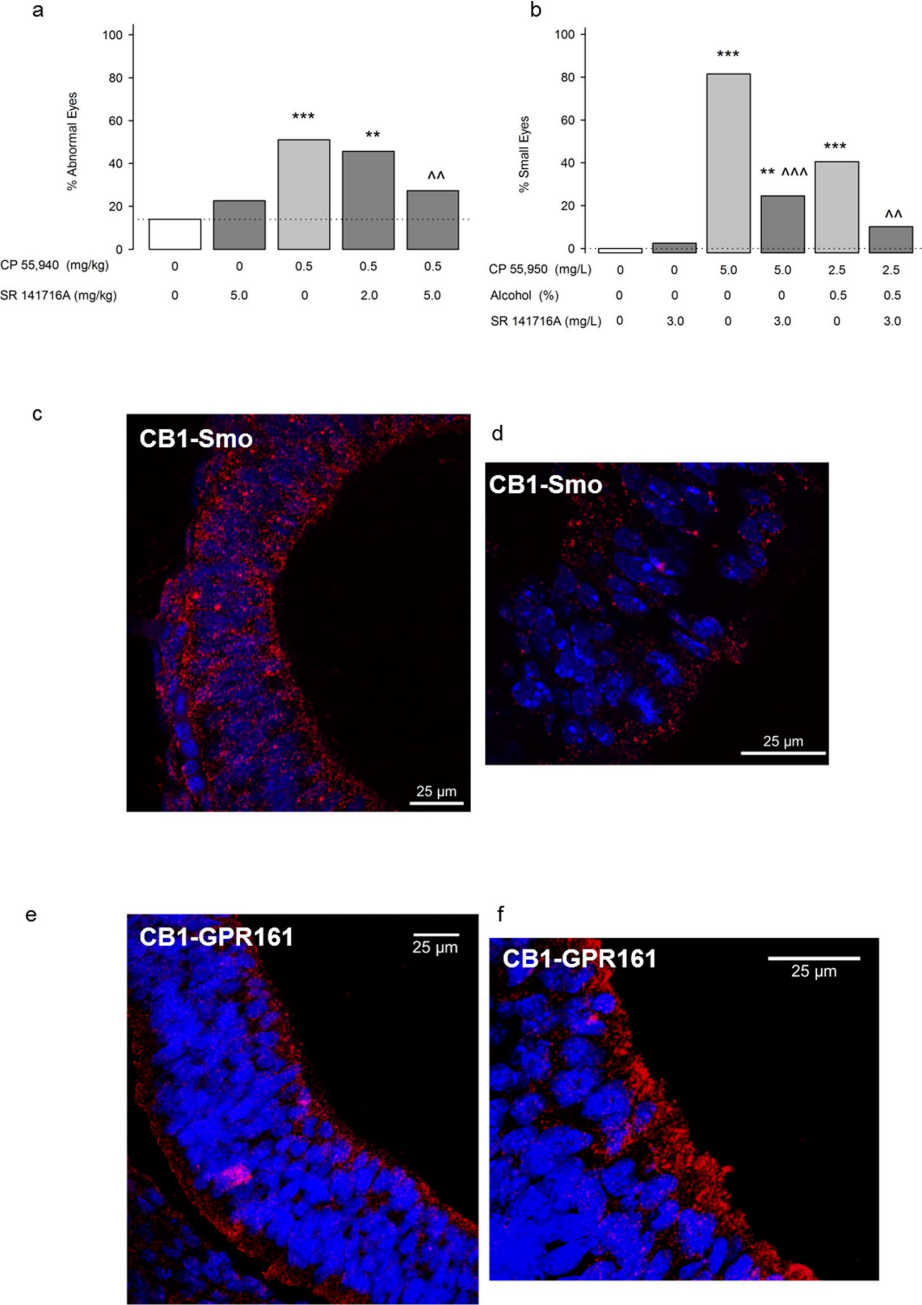
CB1 receptors mediate CB teratogenesis. (**a**) Effects of pretreatment with a CB1 receptor antagonist, SR 141716A, on CP 55,940-induced eye defects in mice. Mice received CB vehicle (n = 43/6 litters) SR 141716A 5.0 mg/kg + the CB vehicle (n = 62/8 litters), the CB vehicle + CP 55,940 0.5 mg/kg (n = 51/8 litters), SR 141716A 2.0 mg/kg + CP 55,940 0.5 mg/kg (n = 45/6 litters), or SR 141716A 5.0 mg/kg + CP 55,940 0.5 mg/kg (n = 88/10 litters). SR 141716A or CB vehicle was given 10 min before the second injection. **p < 0.01, ***p < 0.001 vs. CB vehicle, ^^p < 0.01 vs CP 0.5, using a Chi-square test. Data are the percent of mice with defects (number of affected mice/total number of mice *100). See Supplementary Table 4 for eye defect severity, body weight and length, and the number of live offspring/litter. See Supplementary Fig. 2a–f. for separate analysis of left and right eye defects. (**b**) Small eyes in zebrafish embryos receiving exposure to fish water vehicle (n = 39), SR 141716A 3.0 mg/L (n = 40), CP 55,940 5.0 mg/L alone (n = 54), SR 141716A 3.0 mg/L + CP 55,940 5.0 mg/L alone (n = 49), 0.5% alcohol + CP 55,940 2.5 mg/L (n = 42), SR 141716A 3.0 mg/L + 0.5% alcohol + CP 55,940 2.5 mg/L (n = 49). **p < 0.01, ***p < 0.001 vs. vehicle. ^^p < 0.01, ^^^p < 0.001 vs. corresponding treatment without SR 141716A using a Chi-square test. Data are the percent of zebrafish with defects (number of affected zebrafish/total number of zebrafish *100). (**c,d**) CB1-Smo co-localization in the mouse neural tube (GD 9.5) using a proximity ligation assay (Duolink) to visualize sites (fluorescent red) where anti-CB1 and anti-Smo antibodies bind within 40 nm of one another. (**e,f**). CB1-GPR 161 co-localization in the mouse neural tube (GD 9.5). Nuclei of neural tube cells are labeled with DAPI in blue. Sections (7 µm) were imaged on a confocal microscope using a 40× (**c,e**) and 63× (**d,f**) oil lens.

To explore the role of the CB1 receptor, we tested in mice and zebrafish whether a CB1 receptor antagonist (SR 141716A, Rimonabant) attenuates CP 55,940 teratogenesis. We focused on the CB1 receptor because CB1 is expressed during early neurulation, but CB2 is not detectable^24^. We pretreated mice and zebrafish with SR 141716A before a moderately high dose of CP 55,940 (0.5 mg/kg and 5.0 mg/L in mice and zebrafish, respectively). In zebrafish, we also administered SR 141716A before the combination of a low CP 55,940 dose (2.5 mg/L) and the sub-teratogenic alcohol dose (0.5%) previously found to potentiate each other’s effects (see Fig. 3). Alone, SR 141716A caused a non-significant increase in eye defects, but significantly attenuated CP 55,940-induced eye defects in both species (Fig. 5a,b) and the low fetal weight in the mouse (Supplemental Table 4). SR 141716A also reduced the heightened incidence of eye defects following simultaneous alcohol and CP 55,940 (Fig. 5b). Thus, in addition to Shh signaling, the teratogenic effects of CP 55,940 involve actions upon CB1 receptors.

### CB1 receptors associate with Smoothened

Since our pharmacology studies indicate functional CB1-Shh pathway crosstalk, we explored a potential mechanism for this CB1-Shh interaction. Smo and CB1 are both seven transmembrane protein structures that are primarily linked to inhibitory G-proteins limiting the activation of protein kinase A^55–57^. CB1 and Smo, at least in drosophila, each can form homodimers to maximize their effectiveness at altering cell signaling^58–60^, and CB1 can heterodimerize with many other GPCRs including: CB2^61^; mu^62^ and delta^63^ opioid receptors; 5-HT2A^64^; angiotensin II^65^; orexin^66^; D2^67^; adenosine 2A^68^; and GPR55^69^. In the case of CB1-D2L heteromers, agonist binding can shift G-protein signaling from Gαi to Gαs^70^, and cause an unexpected increase in PKA levels. In the primary cilium, where canonical Shh signaling takes place, PKA activity is reciprocally regulated by Smo and GPR 161^42,56,71^. To ascertain whether CB1 receptors directly associate with Smo, we performed a proximity ligation assay (PLA) in GD 9.5 embryonic neural tube tissue to visualize CB1 and Smo antibodies bound within 40 nm of each other (Fig. 5a,c). Importantly, neither CB1 nor Smo was visualized to interact with negative control proteins expressed in the neural tube; CB1 did not bind to the transcription factor Sox-2, found in the nucleus, and Smo did not interact with Arl13b, which is also a ciliary protein, but does not apparently bind to Smo (Supplementary Fig. 5a,b). However, we did observe that CB1 associated with GPR 161 in a pattern that was similar to CB1-Smo (Fig. 5e). We confirmed the CB1-Smo linkage using co-immunoprecipitation of anti-Smo pull-downs from GD 8 whole embryos and noted that that neither alcohol CP 55,940, nor the simultaneous treatment affected the co-expression of these receptors (Supplementary Fig. 5). These co-immunoprecipitation and PLA data provide the first evidence suggesting that CB1 and Smo form complexes with each other, which may be the targets of prenatal CB exposure.

We hypothesized that if CB1-Smo interactions are potential targets of prenatal alcohol and CB-exposure, then modulation of these interactions would cause G-proteins to dissociate from the CB1-Smo complex. Both CB1 and Smo have been previously shown to associate with Gαi proteins^56,57^, a mechanism for activating Shh signaling. In the co-immunoprecipitation experiment Smo pulled down Gαs; relative to vehicle-treated embryos, all drug treatments reduced the association of Gαs with Smo-CB1. These data reveal that teratogenic exposures alter the coupling of G-proteins, supporting the idea that CB1-Smo complexes are relevant sites of action. This suppression of the Shh pathway leads us to reason that the net signaling of Smo and CB1 is shifted toward stimulating intraciliary cAMP, as has been observed upon activation of CB1-D2L heteromeric receptors^72,73^. It is likely that a similar mechanism is possible in the embryo, where activation of the cannabinoid part of CB1-Smo heteromers may normally limit Shh stimulation. However, CB1-Smo heteromers are also a potential pathogenic mechanism through which excess CB exposure can cause birth defects.

## Discussion

We demonstrate that exposure to phyto- and synthetic CBs during the neurulation stage of embryonic development can cause birth defects like those of prenatal alcohol exposure in mouse and zebrafish models. The exposure periods are the equivalent to the third and fourth weeks of human pregnancy, before most pregnancies are recognized. The similarity between CB-exposed and alcohol-exposed fetuses suggests that some potential CB-induced birth defects may be misidentified as caused by fetal alcohol exposure alone. Our finding that simultaneous exposure to CBs and alcohol induces the highest rate of defects, and that even low doses of alcohol and CBs can potentiate each other’s effects, emphasizes the need for clinical studies to examine fetal outcomes when alcohol and CB exposure occurs at the same time.

When relating these defects to human birth defects, it is important to note the dose-dependency of the synthetic- and the phyto-cannabinoids. Since THC concentrations can vary up to 10–20 fold between cannabis products^17^, and new routes of administration, such as vaporization and “dabbing” of highly concentrated cannabis resins, are emerging^74^, establishing dose-dependency in humans remains a challenge. Over the past decades, as THC concentrations in marijuana have grown, CBD concentrations have diminished considerably^17,75^. Currently however, CBD products are heavily marketed with medicinal claims, and pharmaceutical strength preparations are effective pharmacotherapies for several conditions^50^, making CBD exposure more widespread than it has been previously. We noted that CBD caused eye and midline facial malformations, though overall eye defects were less frequent than with the CB1 receptor agonists. Nonetheless, the teratogenic potential of CBD cautions against the perception that CBD is unequivocally safe. HU-210 and CP 55,940, two CB1 receptor full agonists and members of the large family of synthetic CBs once marketed as legal marijuana alternatives, were potent teratogens. The finding that these synthetic CBs, as well as THC, and CBD exerted similar effects on fetal morphology, in spite of their differing actions at the CB1 receptor, indicates their teratogenic effects involve another mechanism, in addition to the CB1 receptor. Our confirmation of Khaliullina *et al*.^40^ and the *Gli1* and *Shh* mRNA reduction, establish that CBs also inhibit Shh signaling, which may be caused by direct or allosteric modulation of Smo, the primary effector molecule of the Shh pathway. Our discovery of Smo-CB1 receptor heteromers provides the mechanism for an allosteric modulation, though studies in other model systems are required to clarify the function of Smo-CB1 receptors.

Shh pathway alterations have critical developmental effects; impaired activity causes holoprosencephaly spectrum defects, while exaggerated activity causes malformations similar to those observed in some ciliopathies, such as wide faces and brains, and certain cancers^76^. Previous studies demonstrate that alcohol inhibits Shh signaling^25,35,37–39,53^, though precisely how it inhibits Shh is not yet known. Simultaneous alcohol and CB use may be especially damaging to the embryo as the two substances converge mechanistically on the Shh pathway. In zebrafish, the protective effect of *Shh* mRNA on CB teratogenesis, alone or with alcohol, observed for both gross dysmorphology and juvenile behavioral measures^34^, strongly supports Shh signaling inhibition as a pathogenic mechanism. Rather than preventing the binding of CBs to the CB1 receptor and/or to Smo, it is likely that the *Shh* mRNA compensates for the drug-induced inhibition, allowing Shh signaling to normalize. Future studies directly targeting Smo would help address whether the *Shh* mRNA protection occurs downstream of CB1-Smo or through non-canonical actions independent of Smo.

The presence of Smo-CB1 heteromeric receptors in the embryonic neural tube presents a novel mechanism for the regulation of Shh signaling. By forming heteromeric GPCRs, CB1 receptors directly modulate many other receptors and signaling pathways^77^ and can do so through, at least in the case of CB1-D2L receptors, by activating both Gαi and Gαs G-proteins. Our preliminary co-immunoprecipitation data indicate that Smo, which typically associates with Gαi, can also associate with Gαs, presumably when in conjunction with CB1. Interestingly, we also found that CB1 can form a complex with GPR 161, another primary cilia GPCR that associates with Gαs to regulate Shh signaling. Thus, at least some CB1 receptors are localized in the primary cilia to affect Shh signaling through downstream actions on cAMP and PKA^56,71^. PKA regulates the proteolytic processing of Gli transcription factors into their repressor forms; when PKA is stimulated through enhanced Gαs signaling or impaired Gαi signaling, Gli-mediated gene transcription is suppressed.

**Figure 6:**
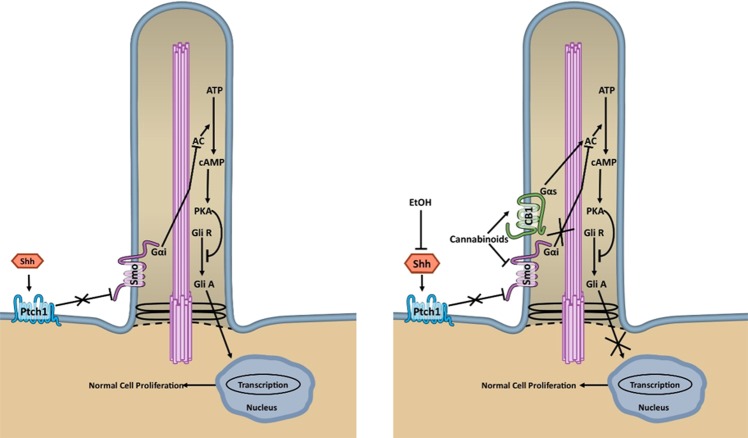
Schematic representation of hypothesized interactions between Smoothened and the CB1 receptor in the embryo. *Left panel*. During typical development, Sonic Hedgehog (Shh) alleviates the repression of Smoothened (Smo) by Patched (Ptch1) which allows Smo to translocate to the primary cilium. In the primary cilium, Smo, a G-protein coupled receptor, activates Shh pathway signaling, partly through its association with Gαi proteins. Gαi inhibits adenyl cyclase (AC) which, by inhibiting the conversion of adenosine triphosphate (ATP) into cyclic adenosine monophosphate (cAMP), inhibits the accumulation of protein kinase A (PKA) and prevents the proteolytic processing of Gli transcription factors into their repressor forms (Gli R). By maintaining the Gli activator (Gli A) state, the inhibition of PKA facilitates the gene transcription necessary for normal cell proliferation and development. *Right panel*. Alcohol (EtOH) and cannabinoids converge onto the Shh pathway to alter typical development. A mechanism by which alcohol affects development is through its inhibition of Shh, which reduces the number of Smo molecules at the primary cilia, and thereby reduces the activation of Shh signaling cascades. Cannabinoids, on the other hand, have two mechanisms of action. First, cannabinoids directly inhibit Smo and prevent the signaling of Smo through Gαi proteins, as described above. Additionally, cannabinoids stimulate CB1 receptors that, in the primary cilia, form heterodimers with Smo. CB1-Smo heterodimers associate with Gαs proteins, in addition to Gαi proteins. When Gαs signaling is activated by CB1 receptor agonism, PKA is stimulated which increases Gli R and decreases Gli A. Co-exposure to alcohol and cannabinoids therefore, inhibits Shh and Smo-Gαi signaling, while simultaneously stimulating CB1-Gαs signaling, and ultimately causing a greater reduction in Shh pathway activation than does exposure to either drug alone.

As illustrated in Fig. 6, we hypothesize that alcohol impairs embryonic development primarily by inhibiting Shh, thereby maintaining the Patched repression of Smo, and preventing normal Smo-Gαi binding and translocation to the primary cilia. The reduction in active Smo molecules restricts the downstream Shh signaling cascades typically induced by Gαi. CBs, on the other hand, directly target active Smo-CB1 molecules, and prevent downstream signaling. By binding to CB1 receptors, CBs increase cAMP production in the primary cilia through Gαs, an effect that may involve GPR161. Together these actions cause excessive proteolytic processing of Gli transcription factors into their repressor forms which subsequently impair downstream Shh signaling. Therefore, we propose that exposure to alcohol and CBs inhibits Shh and Smo-Gαi signaling, stimulates CB1-Gαs signaling, and ultimately reduces Shh pathway activation more than does exposure to either drug alone. These effects are magnified within the primary cilium, the site for Shh signaling, because even small changes in PKA levels within the cilium have large effects on signaling^78,79^.

Overall, our teratology findings should prompt continued thorough evaluations of CBs as teratogens, especially when CBs are used simultaneously with alcohol. Although the interactions described here relate to drug-induced birth defects, it is likely that simultaneous alcohol and CBs users experience similar alcohol-CB interactions on the Shh signaling pathway, which has implications for the effects of these drugs on CNS function, tissue repair and regeneration, and for cancer. Given the overlapping distributions between the Shh and CB1 signaling pathways throughout the lifespan, our finding that CB1 receptors and Smo directly associate, is highly significant. While we demonstrate that Smo-CB1 heteromers are pathogenic targets for prenatal alcohol and CBs, Smo-CB1 heteromers may have roles across development and in multiple disease states. Moreover, compounds that affect Smo-CB1 heteromers have pharmacotherapeutic possibilities and may provide highly specific chemotherapeutics for the treatment of Hh-related cancers.

## Methods

### Animals

Female C57BL/6 J mice (Jackson Labs, Bar Harbor, ME) weighing ca. 20 g were mated to males of the same strain for one to two hrs beginning at least two hrs into the light phase of a 12:12 light:dark schedule. When a copulatory plug was found, gestational day (GD) 0 was designated as the start of the mating session. Mated females were housed in clear, ventilated, polycarbonate cages with no more than three mice per cage, depending on the number of plugs detected in a mating session. Food (Isopro RMH 3000; Purina, St. Louis, MO) and water were freely available, housing and nesting material were provided, and the cob bedding was changed every other week. The vivarium was maintained at 21 ± 1 °C, 30–40% humidity. All mouse experiments followed NIH guidelines using methods approved by the IACUC of UNC at Chapel Hill. Zebrafish were obtained from Zebrafish International Resource Center. The AB strain was used in this study and fish were housed in automatic fish housing systems (Aquaneering, San Diego, CA) at 28.5 °C. All procedures using zebrafish were approved by the North Carolina Central University IACUC and conducted according to NIH and U.S. regulations.

### Cannabinoid teratogenesis

On GD 8, dams were weighed and injected intraperitoneally with freshly prepared drug or vehicle solutions through a 27-gauge needle at a volume of 1.5 ml/100 g body weight. For CB alone studies, the mice received a single injection of either 0.03 or 0.1 mg/kg of HU-210 (3-(1,1′-dimethylheptyl)-6a,7,10,10a-tetrahydro-1-hydroxy-6,6-dimethyl-6H-dibenzo[b,d]pyran-9-methanol), 1.7, 5.6, or 17 mg/kg cannabidiol (2-[1R-3-methyl-6R-(1-methylethenyl)-2-cyclohexen-1-yl]-5-pentyl-1,3-benzenediol), or 0.56, 1.7, 5.6, or 17 mg/kg of Δ^9^-THC (6aR,7,8,10aR-tetrahydro-6,6,9-trimethyl-3-pentyl-6H-dibenzo[b,d]pyran-1-ol. These compounds were obtained from Cayman Chemical (Ann Arbor, MI) and dissolved in deoxygenated 100% ethanol (ethyl alcohol, Pharmaco-Aaper, Brookfield, CT). CB aliquots were prepared immediately before injection by suspension in alkamuls EL 620 (Rhodia, Cranberry, NJ) and dilution to 5% ethanol, 5% alkamuls in lactated Ringer’s solution (i.e. 1:1:18). Control mice were injected with an equivalent volume of the CB vehicle solution, which provides a 0.59 g/kg dose of alcohol and causes peak maternal blood alcohol levels of 43.8 mg/dl^24^.

For the combined CB and alcohol studies, HU-210 (0.03 mg/kg), THC (0.56 mg/kg) or CP 55,940 (0.25 mg/kg, (−)-*cis*-3-[2-hydroxy-4-(1,1-dimethylheptyl)phenyl]-*trans*-4-(3-hydroxypropyl)cyclohexanol, Tocris Bioscience, Minneapolis, MN) suspensions were diluted to 23.7%, 11.85%, or 5% ethanol and 5% alkamuls to administer the 2.8 g/kg, 1.4 g/kg alcohol doses, or the CB vehicle doses, respectively. The mice received a second injection of either the CB vehicle or the appropriate alcohol dose in the CB vehicle, four hrs after the first injection. No additional CB dose was given in the second injection. The purpose of the second injection was to follow established procedures for inducing birth defects following early gestational acute alcohol exposure^3^. Peak blood alcohol levels following the second 2.8 g/kg dose are 381 mg/dl^80^ and 132.2 mg/dl following the 1.4 g/kg dose (Supplemental Table 3). Since there was no difference between mice treated with a single injection of HU-210 (0.03 mg/kg) or THC (0.56 mg/kg) alone (in CB dose-response studies) or mice treated with these doses and a second injection with the CB vehicle (in the alcohol-CB interaction studies), these data were combined.

Blood alcohol levels were measured in GD 8 dams (n = 5/treatment) injected with alcohol (1.4 g/kg) alone, or alcohol combined with CP 55,940 (0.25 mg/kg), and all mice received a second alcohol injection, 4 hrs later. 30μl of tail blood was collected 30 min after the first alcohol injection, and 30, and 60 min after the second alcohol injection. 2 hrs after the second alcohol injection, the mice were euthanized with CO_2_ and cardiac blood was collected. Alcohol levels were measured enzymatically by an Analox alcohol analyzer (Model AM1, Analox Instruments, Lunenburg MA).

For the CB1 receptor antagonist study, SR 141716A (5-(4-chlorophenyl)-1-(2,4-dichlorophenyl)-4-methyl-N-1-piperidinyl-1H-pyrazole-3-carboxamide) was obtained from Tocris Biochemicals (Minneapolis, MN), prepared as above, but diluted to a 5% ethanol, 5% alkamuls suspension in lactated Ringer’s (i.e. 1:1:18). SR 141716A (2.0 or 5.0 mg/kg) or the CB vehicle was administered 10 min before CP 55,940 (0.5 mg/kg) or the CB vehicle. In this experiment, the volume of each injection was 0.75 ml/100 g to ensure that the dams received comparable amounts of alcohol from the CB vehicle (i.e. 0.59 g/kg) as in our other dose-response studies.

Fetal dysmorphology measurements were taken on GD 17. Dams were deeply anesthetized with CO_2_ inhalation and euthanized by cervical dislocation. The uteri were removed, and the fetuses were dissected free under ice-cold phosphate buffered saline (PBS). Fetuses were individually examined for body weight, crown-rump length, gross dysmorphology, and the presence of eye defects, evaluated on a seven-point dysmorphology scale (Fig. 1) based on^24,51^. Fetuses were photographed using a Nikon SMZ-U stereoscopic zoom dissecting microscope (Nikon Corporation, Melville, NY) with a Micropublisher 5.0 digital camera and QCapture Suite software (QImaging, Surrey, BC). After being photographed, the fetuses were immersion fixed in Bouin’s solution or 10% formalin. Select fetuses with eye and/or facial malformations were processed for routine histological analyses. Bouin’s-fixed heads were cleared in 70% ethanol, embedded in paraffin, and sliced coronally at 10 μm using a rotary microtome. Serial sections were mounted on glass slides and stained with hematoxylin and eosin (H&E).

Nile blue sulfate staining, modified from^81^, was performed in embryos treated with the CB vehicle (n = 9 embryos from 3 litters) or 0.5 mg/kg of CP 55,940 (n = 8 embryos from 3 litters) at GD 8. Twelve hrs after the injection (GD 8.5), the embryos were dissected in lactated Ringer’s solution at 37 °C, freed from all extra-embryonic tissues, transferred to a 37 °C Nile Blue solution (1:50,000), and occasionally swirled for 30 min. Following a brief rinse in lactated Ringer’s, the embryos were immediately photographed, as above.

Zebrafish embryos were incubated in 100 mm plates containing 10 to 20 embryos in fish water (60 µg/ml Oceans’ natural sea salt mix dissolved in distilled water; Oceans Reefs & Aquariums, Fort Pierce, Fl) containing a 1:500 dilution of 0.1% methylene blue (to prevent fungal infection). Embryos were exposed from 5.25 to 48 hrs post fertilization (hpf) to CP 55,940 dissolved in DMSO and diluted in fish water to 1 mg/L, 2.5 mg/L, 3.75, or 5 mg/L. DMSO was added to the fish water as control for CP 55,940. Ethanol was diluted in fish water to either 0.5% or 2%. At the end of treatments, fish water containing CP 55,940 and/or ethanol was removed, and embryos were washed once with fresh fish water. Malformation of the MHB was assessed visually, based on the absence of the defined border between the midbrain and hindbrain at 24 hpf stage (see Fig. 3b). Eye size was measured as previously described^82^ and involved measuring the longest axis along the eye, calculated against a standard 50-µm ruler under the same magnification. For the 48 hpf eye, we designated a diameter less than 240 µm as having a small eye phenotype, because untreated eyes were typically at least 250 µm in diameter. All the surviving embryos from different treatments were collected at 24 hpf and/or 48 hpf, and fixed in 4% paraformaldehyde in PBS.

For the ShhN183 mRNA injection study, one to two cell stage embryos were injected with 25 pg of capped ShhN183 mRNA. N-terminal 183 amino acid zebrafish Shh (ShhN183) was synthesized by using 22 hpf zebrafish embryo mRNA as template and oligo dT as primer, using Superscript reverse transcriptase. PCR primers were CGGAATTCATGCGGCTTTTGACGAGAGTG and GCTCTAGATCAGCAATGAATGTGGGCTTTGG. The PCR product was subcloned into pCS2 vector and capped mRNA was synthesized with Ambion cap mRNA kit^82^. For the CB1 receptor antagonist study, SR 141716A was dissolved in DMSO and added to fish water at a concentration of 3 mg/L 30 min before CP 55,940 exposure.

### SHH-L2 and C3H10T1/2 assays of select cannabinoid compounds

#### Compound preparations

Cannabinoid compounds including CP 55,940 (Tocris Biochemicals, Minneapolis, MN), cannabidiol, and HU-210 (both from Cayman Chemical, Ann Arbor, MI) were prepared as 25 mM stocks in deoxygenated DMSO supplemented with 0.1 mg/mL fatty acid-free BSA (A6003; Sigma-Aldrich, St. Louis, MO). The Smoothened agonist, SAG (N-methyl-n′-(3-pyridinlybenzyl)-n′-(3-chlorobenzo[b]thiophen-2-caronyl)-1,4-diaminocyclohexane, Santa Cruz Biotechnology, Dallas, TX) was prepared in DMSO.

#### SHH-L2 Assay for Hedgehog pathway activity

Shh-L2 cells (American Type Culture Collection, Manassas, VA) were plated using the Mutidrop-384 (ThermoFisher) at 3000 cells per well in 384-well plates in complete growth media (DMEM medium Hyclone #SH30243.01, 10% fetal calf serum Hyclone #SH30072.03, 1.5 g/L sodium bicarbonate, GE Healthcare Life Sciences, Pittsburgh, PA). After the cells reached 100% confluency (48 hrs later), the media was changed to induction media (Opti-MEM I reduced serum medium [#31985–070, Invitrogen, Carlsbad, CA], 0.5% fetal calf serum, 1% HyClone non-essential amino acids [#SH3023801, GE Healthcare Life Sciences, Pittsburgh, PA], 1 mM HyClone sodium pyruvate [#SH30239.01, GE Healthcare Life Sciences, Pittsburgh, PA], 10 mM HyClone HEPES Solution [#SH3023701, GE Healthcare Life Sciences, Pittsburgh, PA], and 1% penicillin/streptomycin [#SV30010, GE Healthcare Life Sciences, Pittsburgh, PA]). The Biomek NX workstation (Beckman Coulter Life Sciences, Indianapolis, IN) was used to remove and add media. CB treatments were performed at the following concentrations (5, 10, 15, 20, 30 µM) in the presence of randomized triplicate SAG concentration responses (up to 0.5 µM). For the CP 55,940 concentration response, a maximally effective 300 nM SAG concentration was added to the induction media. Randomized triplicate CB dose responses were then conducted using the D300 digital dispenser (Hewlett-Packard Company, Corvallis, OR). For controls, stimulated compound vehicle wells were included to determine baseline stimulation and ‘no SAG’ wells to calculate fold induction. After 48-hrs incubation with the compounds, 30 µl of the media was removed (20 µl left) and the dual luciferase assay was conducted following a published protocol^83^. First, the firefly luciferase assay buffer was added using the 384-dispense head fluidics (Nanoscreen, North Charleston, SC) and the firefly signal was read using a luminescence plate reader (Pherastar, BMG Labtech, Offenburg, DE). Next, the renilla assay buffer was added to quench the firefly signal and read the renilla signal. Data were expressed as % change in the firefly:renilla ratio from the maximal SAG alone effect, and plotted using a sigmoidal curve fit.

To determine the effects of CBD, CP 55,940, and HU-210 on the viability of the Shh-L2 cells, 384-well plates were seeded with 700 cells per well to a total volume of 45 uL using the Multidrop 384 microplate dispenser and cells were allowed to attach overnight. Cells were treated with CBs or DMSO vehicle control and cultured for a further 72 h. Media was removed and replaced by pre-warmed dye cocktail in PBS (10 ug/mL Hoechst 33342, YOYO-1, 500 nM MitoTracker Red FM). Plates were then returned to the incubator for 45 min, washed and then fixed by adding 20 uL 10% formalin for 15 min. Plates were washed with PBS and then sealed for imaging. Fluorescence quantification and localization was determined using a ThermoFisher CellInsight NXT and 3-channel Cell Health Profiling protocol in HCS Suite software (ThermoFisher). Excitation wavelengths were 386 nm, 485 nm, and 549 nm for Hoechst 33342, YOYO-1, and MitoTracker Red FM, respectively. Fixed exposure times were optimized in each channel for each experiment and set so that camera pixel intensity saturation was not reached. Images were acquired using an Olympus UPlanFLN 10 × /0.30 objective and 2 × 2 camera binning. A nuclear mask was established using channel 1 signaling (Hoechst 33342) and used to determine nuclear characteristics (nuclear count, size, aspect ratio, and texture) as well as establishing the regions of interest for YOYO-1 (area inside nuclear mask; channel 2 mean average intensity) and MitoTracker Red FM (1 pixel eroded into the nuclear mask and expanding 4 pixels outward into cytoplasmic area; channel 3 mean target total intensity for mitochondrial mass and channel 3 mean target average intensity for mitochondrial brightness). Data analysis was performed retrieving data sets from HCS View software (ThermoFisher) and normalizing to mean DMSO values.

#### C3H10T1/2 Alkaline phosphatase assay for Hedgehog pathway activity

C3H10T1/2 cells (CRL-3268; American Type Culture Collection, Manassas, VA) between passages 5 and 15 were plated in 96-well plates at 5000 cells per well from sub-confluent propagation flasks in Dulbecco’s Eagle’s high glucose medium (#SH30081.02; HyClone, South Logan, UT), supplemented with 10% bovine calf serum (#SH30072.03; HyClone, South Logan, UT), 2 mM L-glutamine (HyClone, South Logan, UT), and 100 units/ml penicillin/100 units/ml streptomycin (HyClone, South Logan, UT) following previously published methods^84^. The cells attached overnight resulting in 50% confluency for treatment the day after plating. For CB dose-response studies, cells were stimulated with the previously determined EC_90_ dose of SAG (100 nM). CBs were then added to treatment wells using a D300 digital dispenser (Hewlett-Packard Company, Corvallis, OR) at 1:2 or 1:3 serial dilutions in triplicate. Intra-assay controls included DMSO-treated and SAG-stimulated wells alone, and “no cell” wells for background subtraction. Plates were then sealed with gas permeable, liquid impermeable membranes (#Z380059; Breathe-Easy, Sigma-Aldrich, St. Louis, MO) and incubated under humidified conditions at 37 °C and 5% CO_2_. After 5 days of treatment exposure, the medium was removed from the wells and replaced with a brief 37 °C PBS wash. The PBS was removed and 1X pNPP alkaline phosphatase substrate (#34047; Thermo Scientific, Waltham, MA) in diethanolamine (#34064; Thermo Scientific, Waltham, MA) was added in a volume of 100 µL per well as per the manufacturer’s instructions. Plates were incubated at room temperature in the dark for one hr and colorimetric development was evaluated at 405 nm by SPECTRAmax PLUS 384 (Molecular Devices, Silicon Valley, CA). Data analysis was performed by subtracting “no cell” averages within the same plate from all treatment wells and presenting as percent inhibition vs. DMSO control for plots generated in GraphPad Prism (La Jolla, CA). Plotted data were fit to a sigmoidal curve.

### Shh pathway gene expression assays

Dams were euthanized as above, 24 hrs after injection with either the CB vehicle or CP 55,940 (0.25 mg/kg). The embryos were quickly dissected from the placenta in chilled RNAse-free Dulbecco’s solution. After counting the number of somites for developmental staging, the headfolds were removed and rapidly frozen on dry ice and stored at −80 °C. The tissue was homogenized via vortexing in buffer RLT plus (#1053393, Qiagen Inc., Valencia, CA). RNA was extracted using the RNeasy plus micro kit (#74034, Qiagen Inc., Valencia, CA) and stored at −80 °C. Nucleic acid concentrations and quality were determined using fluorimetry (Qubit 3.0, ThermoScientific, Waltham, MA) and spectrophotometry (NanoDrop 2000, ThermoScientific, Waltham, MA). cDNA was synthesized from the RNA using the Quantitect reverse transcription kit (Qiagen Inc., Valencia, CA). Gene expression was assessed using real-time quantitative PCR (StepOne Plus, Applied Biosystems, Foster City, CA) with Taqman probes (Life Technologies, Carlsbad, CA) for the following target genes: *Shh* (Mm00436528), *Gli1* (Mm00494654), and *Gli2* (Mm01293117). *18 s* (Mm03928990) was used as a reference gene. All reactions for each gene target and reference were run in triplicate. Product specificity of the amplicon was assessed using ethidium bromide-agarose gel electrophoresis. The comparative Ct method was used to obtain the relative fold change in gene expression of experimental (CP) vs. the average of vehicles per plate^85^. Data were transformed and expressed as the log_2_ fold change from the vehicle group.

### Proximity ligation assay (PLA)

On GD 9.5, pregnant C57BL/6 J dams (Jackson Laboratories, Bar Harbor, ME) were euthanized as above and embryos were quickly dissected from the placenta and decidua, placed into 4% paraformaldehyde in 1X phosphate-buffered saline (PBS), and were stored for one week at 4 °C. Somites were counted to stage-match embryos (24–25 somites) for further processing beginning with paraffinization on a Leica TP1020 tissue processor (Leica Camera AG, Wetzlar, Germany); samples were dehydrated with a graded ethanol series, cleared with toluene, and infiltrated with paraffin (Paraplast Plus). Once infiltrated, the embryos were embedded in paraffin and serially sectioned at 7 μm on a microtome. Three slides (4–5 sections/slide) including tissue from the neural tube were chosen and processed using DuoLink PLA Technology (DUO92101, Red Starter Kit; Sigma-Aldrich, St. Louis, MO) according to the manufacturer’s instructions. Briefly, slides were deparaffinized, rehydrated with a graded ethanol series, placed in a heated citric acid buffer for 30 min, blocked with blocking buffer supplied in the kit for 1 hr, then incubated for 36 hr with either an antibody cocktail (1:500 anti-CB1 [rCB1-CT, made in goat; a gift from Dr. Ken Mackie], 1:1000 anti-Smoothened [ab72130, made in rabbit; Abcam, Cambridge, MA] or anti-CB1, 1:500 anti-GPR 161 [Proteintech 13398-1-AP, made in rabbit) in antibody diluent or antibody diluent alone as a no-primary control. Sections were then processed according to the kit’s instructions using the appropriate *in situ* probes. Two control antibody combinations were also used to test the specificity of the probes: (1) CB1 and Sox-2 (1:1000, AB5603, made in rabbit; EMD-Millipore, Burlington, MA), to test whether CB1 antibody labeling, which should in the membrane or cytosol, would proximity label with a nuclear bound transcription factor; and (2) Smo and Arl13b (1:500, clone N295B/66, made in mouse; UC Davis/NIH NeuroMab Facility, Davis, CA), to test whether Smo would proximity label with a primary cilia bound protein. Otherwise, processing was identical to the parameters described above. Embryos were imaged on a Zeiss 880 confocal microscope with a 40x or 63x oil lens.

### Coimmunoprecipitation studies

GD 8 dams were injected with either the CB vehicle (n = 2), 2.8 g/kg alcohol (n = 2), 0.25 mg/kg CP 55,940 alone (n = 2), or with 2.8 g/kg alcohol (n = 2). Four hrs later, they received a second injection of the CB vehicle, or 2.8 g/kg alcohol. 30 min after the second injection, dams were euthanized as described above and whole embryos were collected, pooled as a litter (to achieve adequate protein concentrations for immunoprecipitation), rapidly frozen on dry ice, and stored at −80 °C. The samples were lysed and solubilized in CHAPS buffer (Sigma-Aldrich, St. Louis, MO; 8 mM in 30 mM Tris/HCl, pH 7.4, 5 mM MgCl_2_, 20% glycerol) with protease inhibitor cocktail (# 13786; Sigma-Aldrich, St. Louis, MO) in a ratio of 1:30 (v:v) following methods described previously^55,86,87^. An affinity resin for the anti-CB1R or anti-Smo was prepared by coupling affinity-purified anti-CB1R or anti-Smo antibodies to Affi-Prep-Hz matrix (#153-6060; Bio-Rad Laboratories, Hercules, CA) according to the manufacturer’s instructions. This method binds periodate-oxidized carbohydrate moieties on the antibody heavy chain to hydrazide-activated methacrylate matrix^88^. The CHAPS solubilized proteins (~10 µg/embryo sample) were precleared by mixing with protein A/G coated sepharose bead without any antibody (1:1 volume ratio) for 30 min at 0–4 °C and centrifuged at 1000 × *g* for 5 min. Supernatant was collected as a precleared sample and then mixed with anti-Smo (1:300; #ab236465; Abcam, Cambridge, UK) or anti-CB1R (1:30; #ab23703; Abcam, Cambridge, UK) affinity resin in a total volume of 100 µl (volume adjusted using solubilization buffer) and incubated for 3 hrs at 0–4 °C with constant rotation mixing. Affinity matrix bound immunoprecipitated proteins were spun down (1000 × *g* for 5 min), washed twice with NaCl/Tris (20 mM Tris/HCl, pH 7.4, 140 mM NaCl and 0.1% Tween-20). Immunoprecipitated proteins were eluted from the matrix with 200 µl glycine buffer (100 mM glycine/HCl, pH 2.5) and eluate was immediately neutralized with Tris/HCl (1.5 M, pH 8). The proteins from the neutralized eluate were then precipitated by adding 8 volumes of CHCl_3_/CH_3_OH/H_2_O (1:4:3), dissolved in Laemmli’s sample buffer containing 5 mM EDTA, and heated at 65 °C for 5 min. Proteins were resolved in 10% SDS-PAGE, followed by Western blot and ECL detection, and probed with Gαs antibody (~39–45 kDa; 1:500 for 6 hr; #sc-135914 HRP; Santa Cruz Biotechnologies; Dallas, TX), or anti-CB1R polyclonal (~64–66 kDa; 1:750 overnight; #ab23703; Abcam, Cambridge, UK). Immunoblots were visualized using a chemiluminescent reagent (Western Bright ECL, Advansta, Menlo Park, CA). Bio-Rad GelDoc XR+ along with Image Lab Software 5.2.1 (Bio-Rad Laboratories, Hercules, CA) was used to capture the images. All images were captured using consistent, standard settings and multiple images were captured at 5 sec intervals for 30–40 sec to track the saturation of the bands. Optimum images (without any oversaturation) were analyzed using Image J software^89^.

The following control experiments optimized the immunoprecipitation conditions^55,86,87^. To control for the specificity of the CB1R receptor antibody, Western blots of murine N18 membranes expressing CB1R or rat brain solubilized membrane fractions were probed with rabbit IgG (Sigma-Aldrich, St. Louis, MO) as the primary antibody and did not exhibit bands appearing in the range of CB1R receptors or G proteins. To control for the immunoprecipitation procedure, the washings following immunoprecipitation were run on the gel; no leakage of either CB1R receptor or G-proteins was observed. We performed a mock immunoprecipitation of the extract over agarose that was lacking the attached antibody. No CB1 receptor or G-protein were eluted from the mock matrix material.

### Statistical analysis

The incidence of eye defects in mice and fish was analyzed with Chi-square tests comparing two specified treatments, either to the CB vehicle control or the cannabinoid alone. Litter size, body weights, crown-rump lengths, and blood alcohol levels were analyzed with one-way analysis of variance (ANOVA) tests and post-hoc Bonferroni comparisons vs. the CB vehicle group as appropriate. *Shh* pathway gene expression was analyzed with unpaired t-tests vs. the CB vehicle group. Incidence data are expressed as the proportion of mice or zebrafish with a specific defect (number of affected individuals/number of individuals in that treatment group * 100). All other data are expressed as mean ± SEM except where otherwise stated. Statistical significance was set as an alpha level of 0.05.

## Supplementary information

Supplemental Figures and Tables

## Data Availability

The data supporting these findings are available from the corresponding author upon reasonable request.
