# Supplementary material for: Cannabinoids Exacerbate Alcohol Teratogenesis by a CB1-Hedgehog Interaction
Source: Sci Rep. 2019 Nov 5;9:16057. doi: 10.1038/s41598-019-52336-w (PMC6831672; doi:10.1038/s41598-019-52336-w)
Supplement: Supplementary file 1 — Supplemental Figures and Tables [file 41598_2019_52336_MOESM1_ESM.docx]

**Cannabinoids Exacerbate Alcohol Teratogenesis by a CB1-Hedgehog Interaction**

Eric W. Fish^1^, Laura B. Murdaugh^1^, Chengjin Zhang^2^, Karen E. Boschen^1^, Oswald Boa-Amponsem^2,3^, Haley N. Mendoza-Romero^1^, Michael Tarpley^4^, Lhoucine Chdid^4^, Somnath Mukhopadhyay^2,5^, Gregory J. Cole^2,6^, Kevin P. Williams^4,7^, and Scott E. Parnell*^1,8,9^

^1^Bowles Center for Alcohol Studies, University of North Carolina, Chapel Hill, NC

^2^Julius L. Chambers Biomedical Biotechnology Research Institute, North Carolina Central University, Durham, NC

^3^Integrated Biosciences Program, North Carolina Central University, Durham, NC

^4^Biomanufacturing Research Institute and Technology Enterprise, North Carolina Central University, Durham, NC

^5^Department of Chemistry and Biochemistry, North Carolina Central University, Durham, NC

^6^Department of Biological and Biomedical Sciences, North Carolina Central University, Durham, NC

^7^Department of Pharmaceutical Sciences, BRITE Institute, North Carolina Central University, Durham, NC

^8^Department of Cell Biology and Physiology, University of North Carolina, Chapel Hill, NC

^9^Carolina Institute for Developmental Disabilities, University of North Carolina, Chapel Hill, NC

***Correspondence to: Scott E. Parnell, Bowles Center for Alcohol Studies, Thurston-Bowles CB# 7178, UNC- Chapel Hill, Chapel Hill, NC 27599

E-mail: sparnell@med.unc.edu


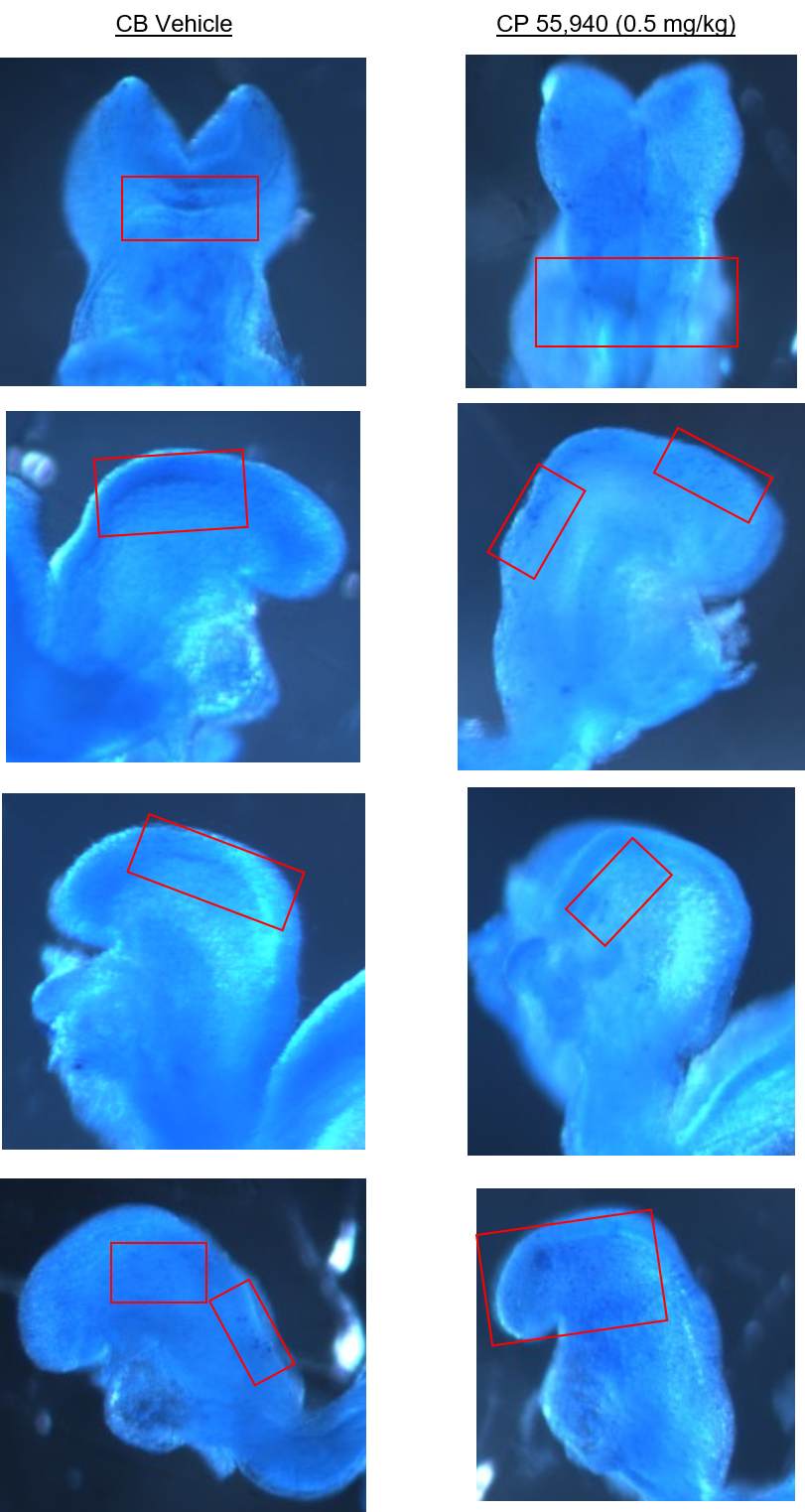


**Supplemental Figure 1.** Nile Blue staining of cannabinoid treated embryos. On GD 8, mice received injections of either the CB vehicle (n=3 litters) or the synthetic cannabinoid CP 55,940 (0.5 mg/kg, n=3 litters) and were processed for Nile Blue staining 12 hours later. Areas of cells death are indicated as dark blue staining within the regions marked by red rectangles. Although the teratogenic effects of CP 55,940 appeared to be similar to those of alcohol, unlike alcohol exposure, an equally teratogenic dose of CP 55,940 (0.5 mg/kg) was not associated with increased cell death relative to the vehicle treatment. Representative embryos are shown.


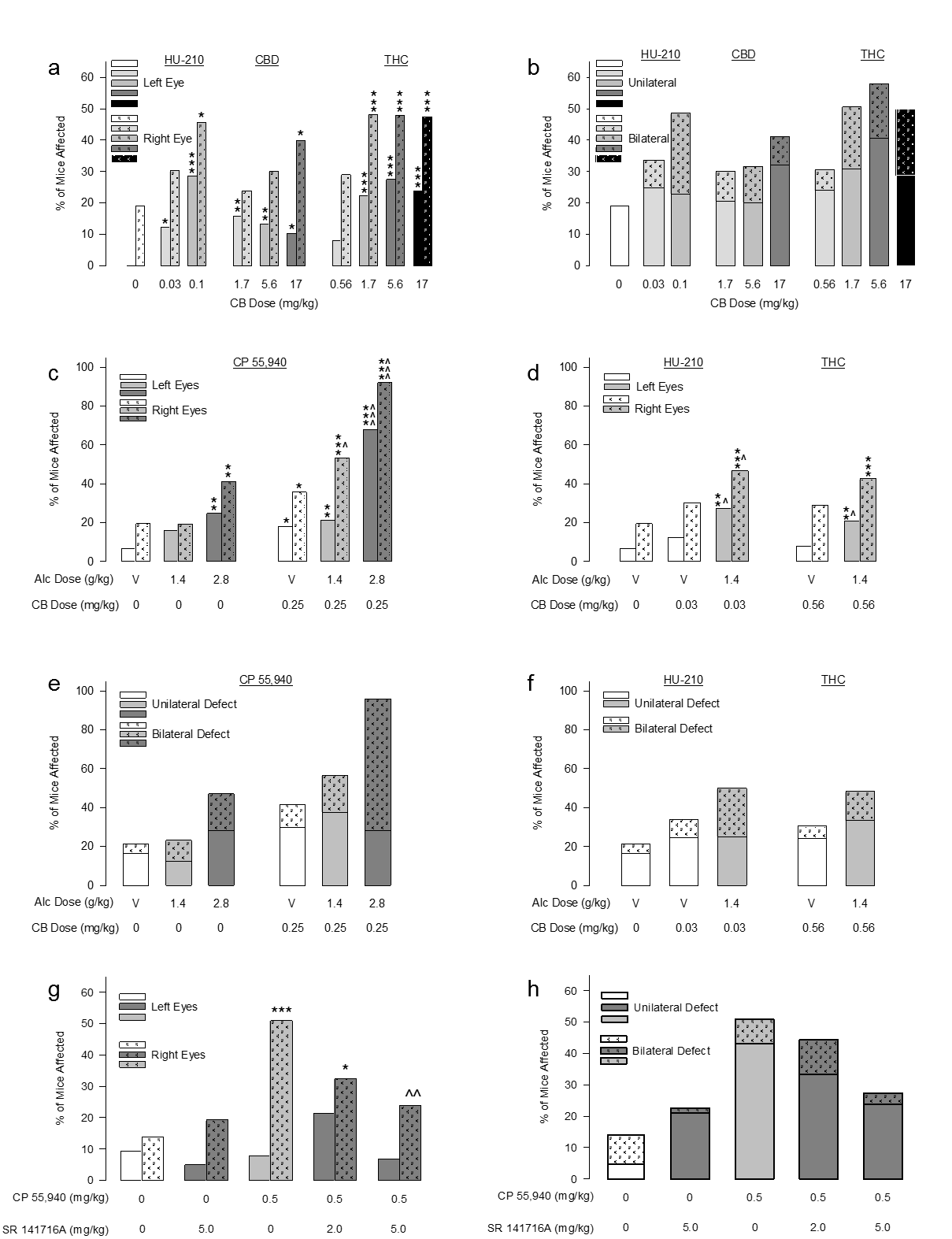


**Supplemental Figure 2.** Prenatal cannabinoid exposure affects both the left and right eyes in fetal mice. (a) The incidence of left (plain bars) and right (dotted bars) eye defects in fetal mice following GD 8 exposure to the CB vehicle (n=63/8 litters), HU-210 0.03 mg/kg (n=89/11 litters), HU-210 0.1mg/kg (n=35/6 litters), CBD 1.7mg/kg (n=63/8 litters), CBD 5.6mg/kg (n=60/7 litters), CBD 17mg/kg (n=78/10 litters), THC 0.56mg/kg (n=76/9 litters), THC 1.7mg/kg (n=81/11 litters), THC 5.6mg/kg (n=69/10 litters), THC 17mg/kg (n=80/10 litters). *p<0.05, **p<0.01, ***p<0.001, Chi-Square vs. CB vehicle with the left and right eyes analyzed separately. (b) Portrayal of unilateral (plain bars) and bilateral defects (dotted bars) from each of the CB treatments. (c&d) Cannabinoid exacerbation of alcohol-induced left and right eye defects in fetal mice following GD 8 exposure to the CB vehicle (n=103/13 litters), Alcohol 1.4g/kg alone (n=62/10 litters), Alcohol 2.8g/kg alone (n=85/10 litters), CP 55,940 0.25mg/kg alone (n=101/12 litters), simultaneous CP 55,940 0.25mg/kg + Alcohol 1.4g/kg (n=133/16 litters), or simultaneous CP 55,940 0.25mg/kg + Alcohol 2.8g/kg (n=50/7 litters). ^p<0.05, ^^^p<0.001 vs. respective doses of CP 55,940 alone using a Chi-square test for the left and right eyes analyzed separately. (e&f) Portrayal of unilateral and bilateral defects from each of the CB-Alcohol treatments. (g) Effects of pretreatment with a CB1 receptor antagonist, SR 141716A, on CP 55,940-induced eye left and right defects in fetal mice. Mice received CB vehicle (n=43/6 litters) SR 1417116A 5.0mg/kg + the CB vehicle (n=62/8 litters), the CB vehicle + CP 55,940 0.5mg/kg (n=51/8 litters), SR 141716A 2.0mg/kg + CP 55,940 0.5mg/kg (n=45/6 litters), or SR 141716A 5.0mg/kg + CP 55,940 0.5mg/kg (n=88/10 litters). SR 141716A or CB vehicle was given 10 min before the second injection. ^^p<0.01 vs CP 0.5, Chi-square test for the left and right eyes analyzed separately. (h) Portrayal of unilateral and bilateral defects from each of the CP 55,940 and SR 141716A treatments. Data are the percent of mice with a left or right-sided defect, a unilateral defect, or a bilateral defect (number of affected mice/total number of mice *100).


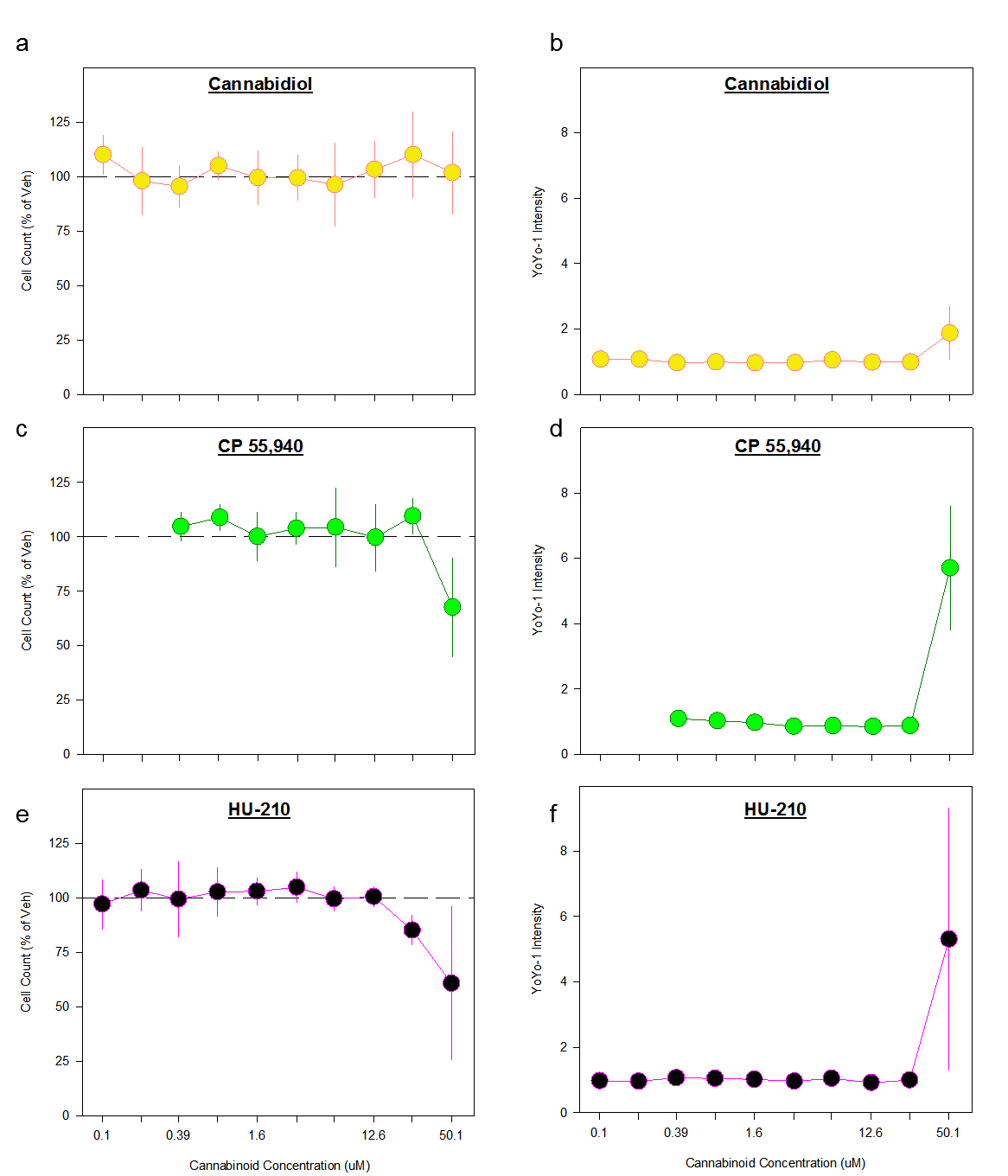


**Supplemental Figure 3.** Effects of CBs on the viability of Shh-L2 cells. Cells were exposed to various concentrations of CBD (a,b), CP 55,940 (c,d), HU-210 (e,f), or DMSO vehicle and then stained with Hoescht 33342 (a,c,e) and YoYo-1 (b,d,f) to determine cell count and activity, respectively. Data are the mean + SEM of four replicates, expressed as a percent of the vehicle (Hoescht staining) or average fluorescent intensity (YoYo-1).


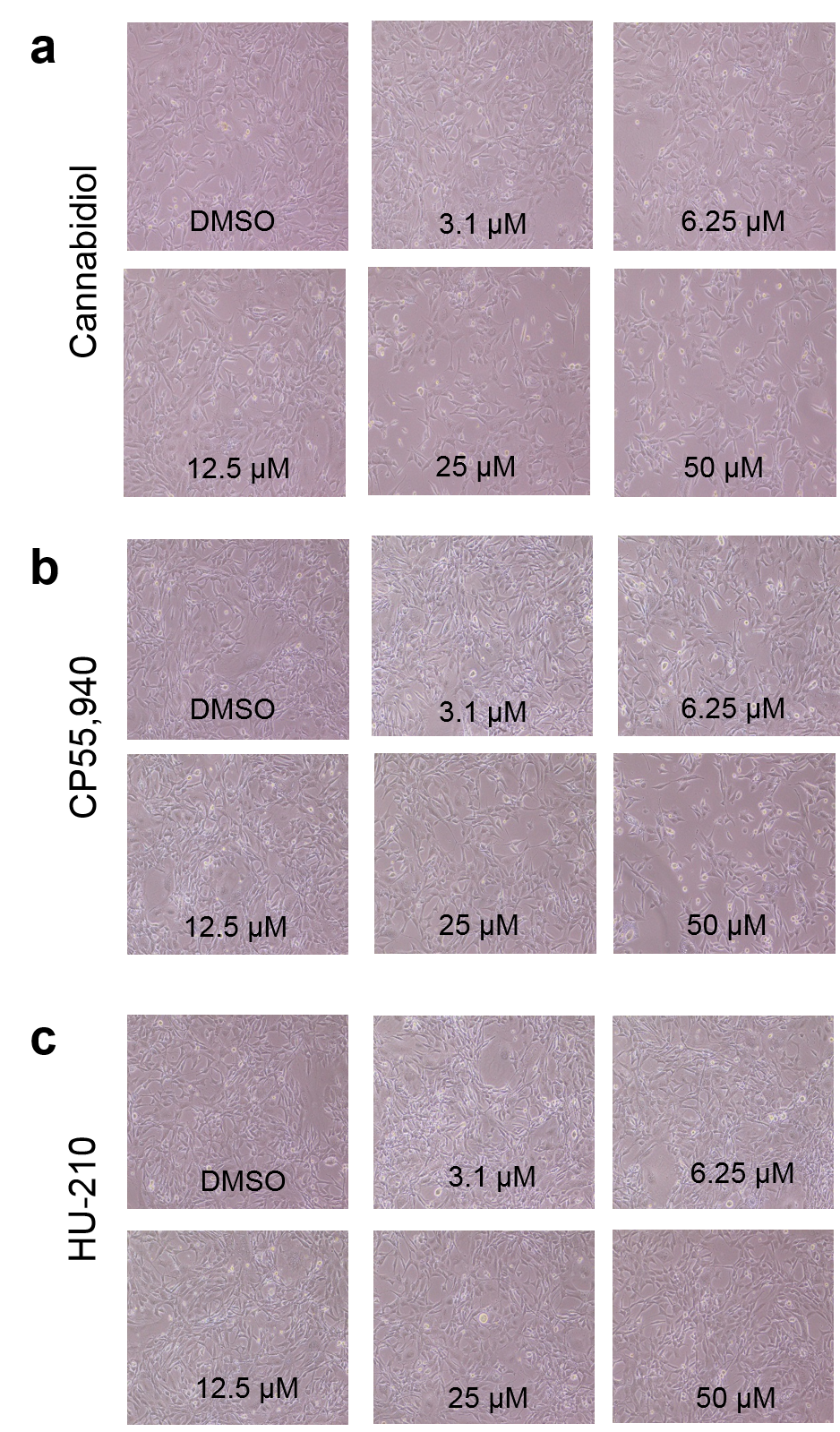
**Supplemental Figure 4.** Effects of CBs on the viability of Shh-L2 cells. Cells were exposed to various concentrations of CBD (a), CP 55,940 (b), HU-210 (c), or DMSO vehicle and then stained with Hoescht 33342. Representative images of cells are shown.


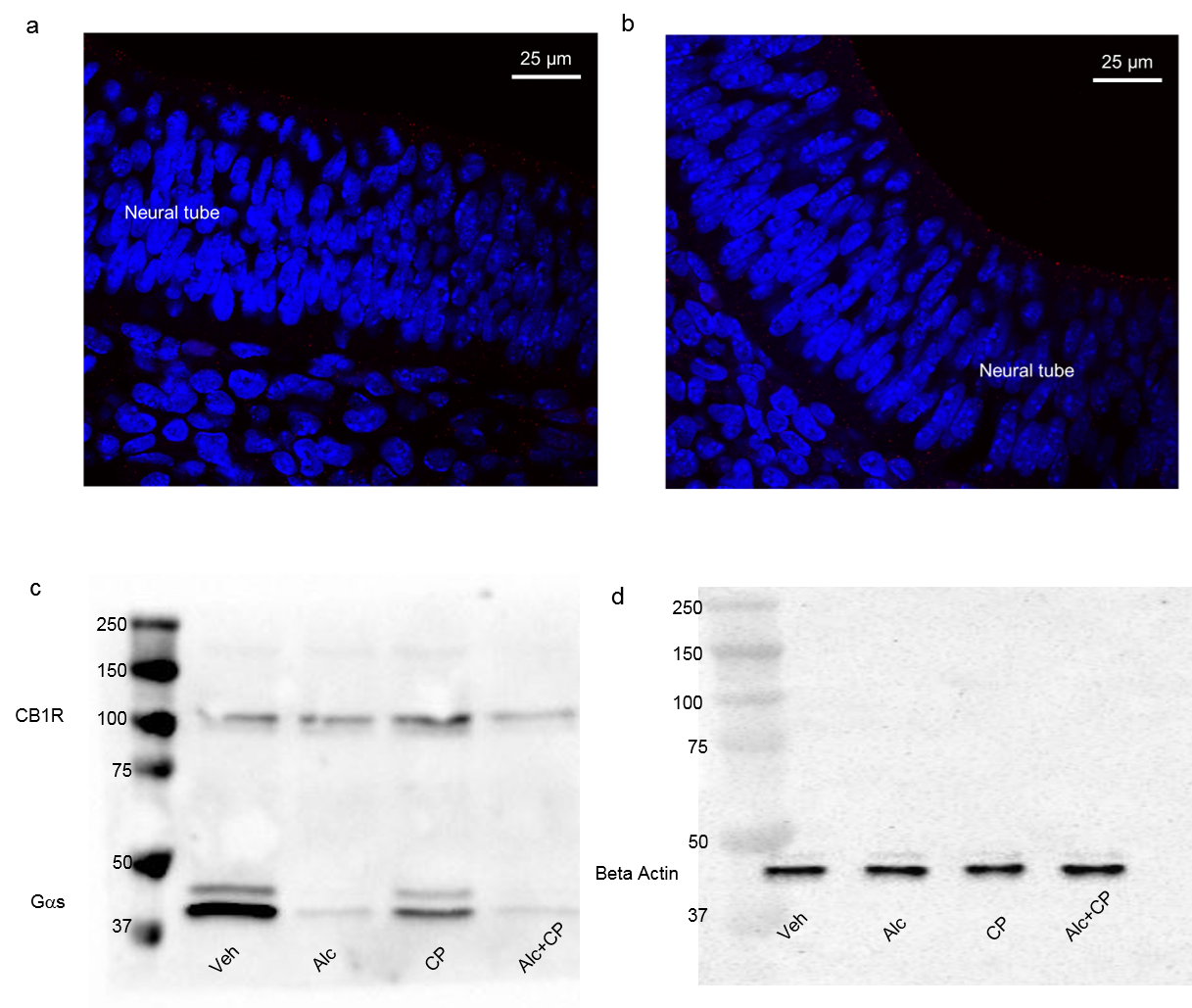


**Supplemental Figure 5.** CB1-Smo protein interactions. (a&b) Negative controls for the proximity ligation assay in the neural tube of GD 9.5 embryos. Sections (7 µm) were processed to determine specificity of the technique using antibody cocktails of (a) anti-CB1 (membrane/cytosol) and anti-Sox-2 (nucleus) and (b) anti-Smo (membrane/cilia) and anti-Arl13b (cilia). Nuclei were stained with DAPI (blue). Very limited fluorescence was seen with either antibody combination (red) and likely represents background signal. In comparison, the fluorescent signal present in the CB1-Smo antibody combination was much stronger (Fig. 5). (c) Co-immunoprecipitation of Smoothened with CB1 and Gαs in whole embryo samples taken 4.5 hrs after GD 8 exposure to either the CB vehicle (Lane 1), 2.8 g/kg alcohol (Lane 2), 0.25 mg/kg CP 55,940 (Lane 3), or the two drugs combined (Lane 4). (d) Loading control for the co-immunoprecipitation experiment using an antibody to detect beta-actin following solubilization.

Supplemental Table 1. Effects GD 8 Cannabinoid Exposure on the Severity of Eye Defects, Body Weight, Body Length, and the Number of Live Fetuses/Litter

Eye Defect Severity Score

Drug Dose (mg/kg) 1 2 3 4 5 6 7 Body Weight Body Length Fetuses/Litter

Veh 51 8 4 0 0 0 0 0.83±0.01 17.4±0.01 7.9±0.40

HU-210 (0.03) 59 29 1 0 0 0 0 **0.76±0.01***** 17.1±0.01 8.1±0.37

HU-210 (0.1) 18 12 3 1 0 0 1 **0.71±0.01***** 17.0±0.01 5.8±1.9

CBD (1.7) 44 16 1 1 1 0 0 **0.79±0.01***** 17.4±0.01 7.9±0.40

CBD (5.6) 41 17 1 1 0 0 0 0.81±0.01 17.8±0.01 8.5±0.67

CBD (17.0) 46 23 4 3 2 0 0 **0.77±0.01***** 17.4±0.01 7.8±0.55

THC (0.56) 53 20 1 0 1 0 1 0.81±0.01 17.8±0.01 8.4±0.48

THC (1.7) 40 38 2 1 0 0 0 **0.77±0.01***** **16.9±0.01***** 7.4±0.38

THC (5.6) 29 40 0 0 0 0 0 **0.74±0.01***** 17.1±0.01 7.1±0.65

THC (17.0) 40 38 2 0 0 0 0 **0.73±0.01***** **16.9±0.01***** 8.0±0.70

Data for eye defect severity are expressed as number of fetuses per eye score. Body weight, body length and number of fetuses/litter are expressed as mean (+ 1SEM). Emboldened data indicate significance vs. Veh (*** p<0.001 following a one-way ANOVA).

Supplemental Table 2. Effects of GD 8 Cannabinoid and Alcohol Co-Exposure on the Severity of Eye Defects, Body Weight, Body Length, and the Number of Live Fetuses/Litter

Eye Defect Severity Score

Drug Treatment 1 2 3 4 5 6 7 Body Weight Body Length Fetuses/Litter

Veh 81 17 1 2 1 1 0 0.79±0.01 16.7±0.09 7.9±0.38

Alc(1.4) 47 13 0 1 1 0 0 0.78±0.01 16.9±0.1 6.2±0.61

Alc(2.8) 45 36 2 0 0 2 0 **0.73±0.01***** 16.9±0.07 7.0±0.82

CP(0.25) 59 40 1 1 0 0 0 0.77±0.01 **16.1±0.07*** 8.4±0.45

Alc(1.4) + CP(0.25) 58 64 8 2 0 0 1 0.77±0.01 16.9±0.08 8.4±0.26

Alc(2.8) + CP(0.25) 2 29 13 2 2 1 1 0.78±0.01 16.9±0.09 6.5±0.60

HU(0.03) 59 29 1 0 0 0 0 0.76±0.01 **17.1±0.01**** 8.1±0.37

Alc(1.4) + HU(0.03) 62 47 6 2 2 2 1 **0.76±0.01*** **17.2±0.01***** 8.1±0.52

THC(0.56) 53 20 1 0 1 0 1 0.80±0.01 **17.8±0.01***** 8.4±0.38

Alc(1.4) + THC(0.56) 45 38 2 2 0 0 0 **0.76±0.01*** **17.1±0.01**** 7.9±0.61

Data for eye defect severity are expressed as number of fetuses per eye score. Body weight, body length and number of fetuses/litter are expressed as mean (+ 1SEM). Emboldened data with asterisks indicate significance vs. Veh (* p<0.05, ** p<0.01, *** p<0.001 following a one-way ANOVA).

Supplemental Table 3. Blood Alcohol Concentrations Following 1.4 g/kg Alcohol in Mice Treated with CB Vehicle or 0.25 mg/kg CP 55,940.

Alcohol Injection 1 Alcohol Injection 2

Time after Alcohol (hours) 0.5 0.5 1.0 2.0

Veh 138.6±5.4 132.2±5.8 93.1±2.9 7.4±3.0

CP 55,940 (0.25) 139.6±2.4 137.6±2.6 101.9±3.9 19.0±4.4

All data are expressed as mean (± SEM). There were no significant effects of CP 55,940 on blood alcohol concentrations.

Supplemental Table 4. Effects GD 8 Exposure to SR 141716A and CP 55,940 Exposure on the Severity of Eye Defects, Body Weight, Body Length, and the Number of Live Fetuses/Litter

Eye Defect Severity Score

Drug Dose (mg/kg) 1 2 3 4 5 6 7 Body Weight Body Length Fetuses/Litter

Veh 37 5 1 0 0 0 0 0.74±0.01 17.2±0.01 7.2±0.8

SR (5.0) 48 11 3 0 0 0 0 **0.78±0.01**** **17.8±0.01*** 7.8±1.0

CP (0.5) 25 18 5 2 0 1 0 **0.71±0.01*** 17.2±0.02 6.4±1.2

SR (2.0) + CP (0.5) 25 18 2 0 0 0 0 **0.71±0.01*** 17.0±0.01 7.5±0.67

SR (5.0) + CP (0.5) 64 20 3 0 1 0 0 **0.79±0.01**** 17.3±0.01 8.8±0.94

Data for eye defect severity are expressed as number of fetuses per eye score. Body weight, body length and number of fetuses/litter are expressed as mean (+ 1SEM). Emboldened data indicate significance vs. Veh (* p<0.05, ** p<0.01, following a one-way ANOVA).
